# Synthesis of Novel Baicalein Amino Acid Derivatives and Biological Evaluation as Neuroprotective Agents

**DOI:** 10.3390/molecules24203647

**Published:** 2019-10-09

**Authors:** Xiaohui Jia, Menglu Jia, Yuqin Yang, Di Wang, Fei Zhou, Wenxi Zhang, Xuemei Huang, Wenbo Guo, Desheng Cai, Hongshan Chen, Jinchai Qi, Shuqi Zhou, Haomiao Ren, Bing Xu, Tao Ma, Penglong Wang, Haimin Lei

**Affiliations:** 1 School of Chinese Pharmacy, Beijing University of Chinese Medicine, Beijing 100102, China; 18811508865@163.com (X.J.); yangyq5@163.com (Y.Y.); zf116318@163.com (F.Z.); zhangwxnn@126.com (W.Z.); hxm3928@163.com (X.H.); wb_guo@126.com (W.G.); cai1225366978@163.com (D.C.); chs1314as@163.com (H.C.); 17862969559@163.com (J.Q.); 18811189872@163.com (H.R.); 2 Beijing Shijingshan District Medical Security Bureau, 66 Yangzhuang Road, Shijingshan, Beijing 100043, China; 15001290933@163.com; 3 Fuxin Health School, Liaoning 123000, China; Diven0804@163.com

**Keywords:** baicalein, neuroprotective effect, amino acids derivatives, SH-SY5Y cell, chick chorioallantoic membrane

## Abstract

Baicalein, a famously effective component of the traditional Chinese medicine Rhizoma Huang Qin (*Scutellaria altissima* L.), has been proved to have potent neuroprotection and anti-platelet aggregation effects with few side effects. Meanwhile, recent studies have revealed that the introduction of amino acid to baicalein could improve its neuroprotective activity. In the present study, a series of novel baicalein amino acid derivatives were designed, synthesized, and screened for their neuroprotective effect against *tert*-butyl, hydroperoxide-induced, SH-SY5Y neurotoxicity cells and toxicity on the normal H9C2 cell line by standard methylthiazol tetrazolium (MTT) assay. In addition, all of the newly synthesized compounds were characterized by ^1^H-NMR, ^13^C-NMR, and high resolution mass spectrometry (HR-MS). The results showed that most of the compounds provided more potent neuroprotection than baicalein, and were equivalent to the positive drug edaravin. They showed no obvious cytotoxicity on normal H9C2 cells. Notably, the most active compound **8** displayed the highest protective effect (50% effective concentration (EC_50_) = 4.31 μM) against *tert*-butyl, hydroperoxide-induced, SH-SY5Y neurotoxicity cells, which was much better than the baicalein (EC_50_ = 24.77 μM) and edaravin (EC_50_ = 5.62 μM). Further research on the chick chorioallantoic membrane (CAM) model indicated that compound **8** could significantly increase angiogenesis, which might promote neurovascular proliferation. The detection of apoptosis analysis showed that compound **8** could dramatically alleviate morphological manifestations of cell damage. Moreover, the benzyloxycarbonyl (cbz)-protected baicalein amino acid derivatives showed better neuroprotective activity than the *t*-Butyloxy carbonyl (boc)-protected derivatives.

## 1. Introduction

Neurodegenerative diseases have become some of the most challenging diseases, which has attracted the attention of scientists all over the world in recent years [1,2,3,4,5]. Alzheimer’s disease (AD), Parkinson’s disease (PD), and other serious neurodegenerative diseases pose a great threat to human life and health [6,7]. Currently, a great deal of treatment techniques and drugs have been developed to treat them, such as dopaminergic treatments, antipsychotic drugs, and brain stimulation [8,9,10,11]. However, the severe side effects of these drugs have greatly hindered their further clinical application [12,13]. Therefore, the search for neuroprotective drugs with high efficiency and low toxicity to treat neurodegenerative diseases from natural resources is an important research direction in the future [14,15,16,17,18].

Baicalein (5,6,7-trihydroxyflavone), a major and famous effective component of the traditional Chinese medicine Rhizoma Huang Qin (*Scutellaria altissima* L.), has been proved to have therapeutic effects against Alzheimer’s and Parkinson’s diseases [19,20,21,22]; it has been expected to be an ideal drug for the treatment of neurodegenerative diseases. However, its clinical application has been greatly limited by poor oral bioavailability [23,24,25,26]. Recent studies revealed that amino acids showed unique neurotransmitter effects in the central nervous system and could change the lipid–water partition coefficient [27,28,29,30,31,32,33]. In addition, some amino acids not only guarantee the normal development of the body, but also improve the function of central nervous tissue, such as l-type lysine [34]. These have inspired interest in using baicalein as the template parent to synthesize novel neuroprotective agents by combination with amino acids. Moreover, many other studies have indicated that in baicalein derivatives, the hydroxyl group remains at the C-5 position, and has a strong activity after being modified by C-6 or C-7 [35]. Based on the above, a series of baicalein amino acid derivatives had been designed and synthesized to improve baicalein’s neuroprotective effects. In addition, all of the newly synthesized compounds were characterized by ^1^H-NMR, ^13^C-NMR, and high resolution mass spectrometry (HR-MS), and tested for neuroprotective activity against SH-SY5Y (liver neurons), and H9C2 (human cardiac myocytes) cell lines. Moreover, the most selectivity compound was investigated by the chick chorioallantoic membrane (CAM) test and fluorescence staining observation. The structure–activity relationships of these derivatives are also briefly discussed. Remarkably, the current findings suggest that compound **8** could not only effectively protect the damaged nerve cell, but also promote angiogenesis, providing a novel effective treatment strategy for the following study of derivative baicaleins.

## 2. Results

### 2.1. Chemistry

**Scheme 1:**
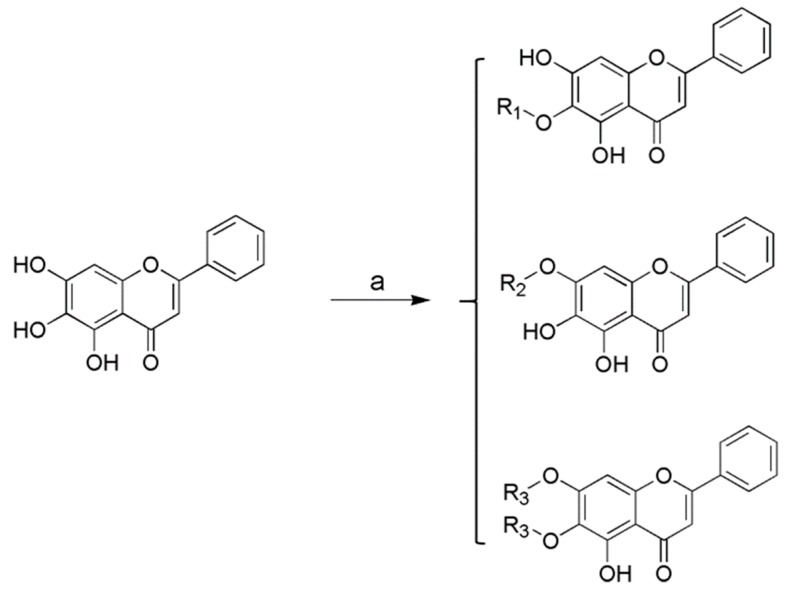

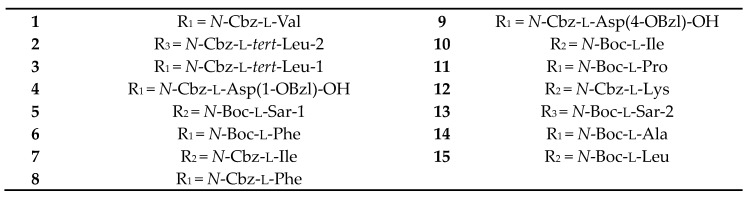
Synthesis of the baicalein derivatives (**1**–**15**). Reagents and conditions: (a) boc-amino acids or cbz-amino acids, dichloromethane (DCM), 4-dimethylaminopyridine (DMAP), and 1-Ethyl-3-(3-dimethylaminopropyl) carbodiimide hydrochloride (EDCI); 25 °C; 12 h.

As shown in Scheme 1, all the designed derivatives were synthesized following the procedures. In Scheme 1, compounds **1**–**15** were produced by 1-Ethyl-3-(3-dimethylaminopropyl) carbodiimide hydrochloride (EDCI)- and 4-dimethylaminopyridine (DMAP)-mediated esterification from the corresponding protected (*t*-Butyloxy carbonyl, benzyloxycarbonyl) amino acids and baicalein. The structures of all target derivatives are shown in Table 1, and all of them were confirmed by spectral (^1^H-NMR, ^13^C-NMR, and HRMS) analysis.

### 2.2. Biological Activities

#### 2.2.1. Protective Effects against *Tert*-Utyl, Hydroperoxide-Induced, SH-SY5Y Neurotoxicity Cells

**Figure 1:**
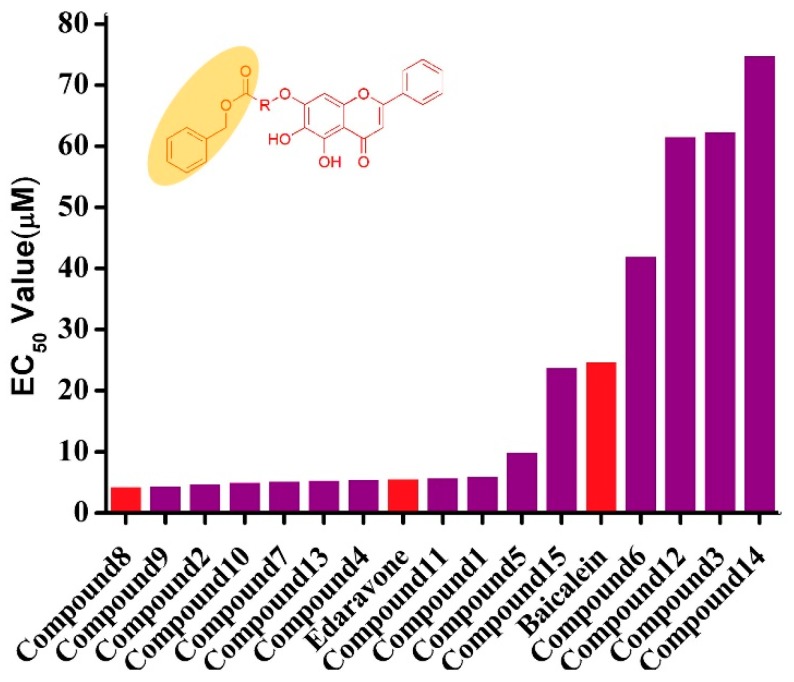
Structure–activity relationships of baicalein derivatives. The results show that cbz-protected baicalein amino acid derivatives show better neuroprotective activity than the boc-protected baicalein amino acid derivatives.

The protective effects of baicalein derivatives in vitro was evaluated against *tert*-butyl, hydroperoxide-induced, SH-SY5Y neurotoxicity cells using an MTT assay. In addition, their toxicity evaluation was tested on normal H9C2 cells. The resulting proliferation rates (%) at different concentrations, as well as their 50% effective concentrations (EC_50_) for protecting damaged SH-SY5Y cells of the baicalein amino acid derivatives, are summarized in Table 2. The results showed that baicalein exhibited protective effects on injured SH-SY5Y cells, and most of the compounds were more potent (with lower EC_50_ values) against injured SH-SY5Y cells than the positive baicalein drugs (EC_50_ = 24.77 µM). The protective effects of several compounds were similar to the widely used neuroprotective drugs edaravone (EC_50_ = 5.62 µM). Among all the compounds, compound **8** was the most active, with an EC_50_ value at 4.31 μM. The structure–activity relationships showed that the amino acid substituent at the 6-OH position exhibited much better neuroprotective activities than other amino acid derivatives, in the sequence **8** > **9** > **2** > **10** (Figure 1).

Moreover, most of the cbz-protected baicalein amino acid derivatives (**2**, **7**, **8**, **9**) showed better neuroprotective activity than the boc-protected baicalein amino acid derivatives (**5**, **6**, **11**, **15**). The current study also showed that compound **8** exhibited good neuroprotective activities. To study cytotoxicity on normal cells, the cytotoxicity of compounds against normal H9C2 cells were tested. As shown in Table 3, the results indicate that there was almost no toxicity against H9C2 cells of all the compounds at 3.125 µM to 50 µM.

Based on the above evidence, we reason that the new synthetic baicalein derivatives possess remarkable neuroprotective activities. These results may provide a new idea for the design of baicalein derivatives with neuroprotective drugs to treat neurological diseases.

#### 2.2.2. Analyses of Apoptosis

Apoptosis has been considered as the main mechanism of chemotherapy-induced cell death. Also, apoptosis can be differentiated from necrosis by their characteristic nuclear changes. Thus, we preliminarily studied the mechanism of action of compound **8** via the Giemsa staining method [36]. Then we studied the mechanism of action of compound **8** on injured SH-SY5Y cells through the detection of apoptosis, using DAPI staining.

##### Morphological Detection of Apoptosis Using Giemsa Staining

**Figure 2:**
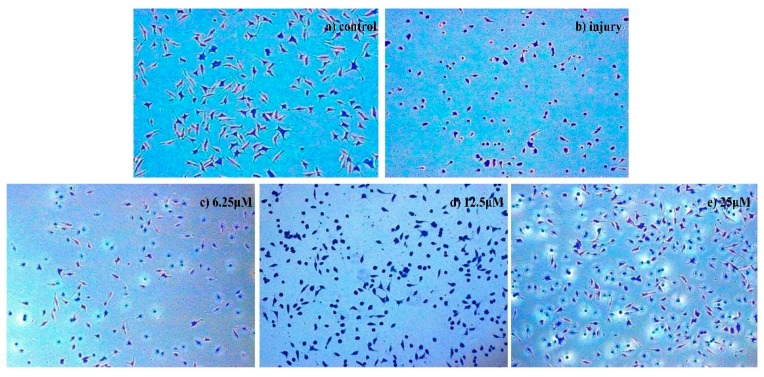
Morphological detection of apoptosis using Giemsa staining (200×) on injured SH-SY5Y cells treated with compound **8**: (**a**) control group, (**b**) injury group, (**c**) 6.25 μM, (**d**) 12.5 μM, and (**e**) 25 μM.

To characterize the morphological detection of apoptosis induced by compound **8** on injured SH-SY5Y cells, the nuclear and cytoplasmic morphological changes in compound **8**-treated injured SH-SY5Y cells were observed with Giemsa staining. As shown in Figure 2, the number of injured SH-SY5Y cells in the control group was much higher than in the injury groups, and the cells in the injury groups appeared to show shrinkage and disruption, with an unusual cell shape. With the dose increase, the phenomenon of cell shrinkage and cell disruption became less obvious, alleviating morphological manifestations of cell damage compared to model cells.

##### Morphological Detection of Apoptosis Using DAPI Staining

**Figure 3:**
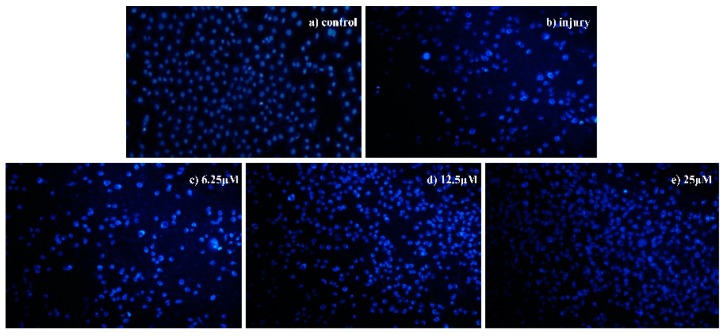
Morphological detection of apoptosis using 4′,6-diamidino-2-phenylindole (DAPI) staining (200×) on injured SH-SY5Y cells treated with compound **8**, (**a**) control group, (**b**) injury group, (**c**) 6.25 μM, (**d**) 12.5 μM, and (**e**) 25 μM.

To further study the mechanism of growth inhibition of compound **8** on injured SH-SY5Y cells, DAPI staining was performed. Injured SH-SY5Y cells were treated with compound **8** at 6.25, 12.5, and 5 μM concentrations for 72 h. As shown in Figure 3, for compound **8**, control groups of SH-SY5Y cells showed intact cell bodies with clear round nuclei, and nuclear staining was slightly blue, while in the injury groups, nuclear morphological changes were typical of apoptosis, the number of cells was decreased, and nuclear condensation, nuclear fragmentation, and the formation of apoptotic bodies appeared. When the concentration of the drug increased, the shape of the cells became regular. Thus, the results indicated that compound **8** could protects injured SH-SY5Y cells from apoptosis.

#### 2.2.3. Angiogenesis Activity

**Figure 4:**
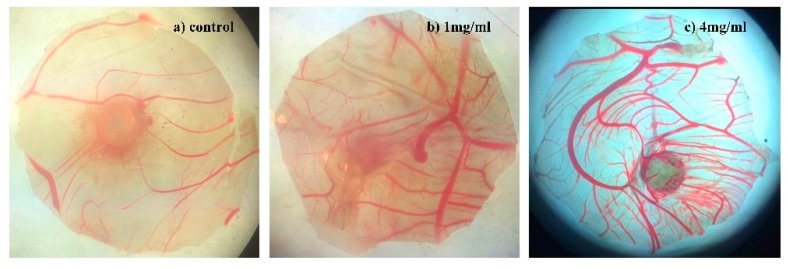
Microvascular proliferation of compound **8** on chick chorioallantoic membrane (CAM) (×50). (**a**) Blank control group, (**b**) 1 mg/mL for compound **8** group, and (**c**) 4 mg/mL for compound **8** group.

**Figure 5:**
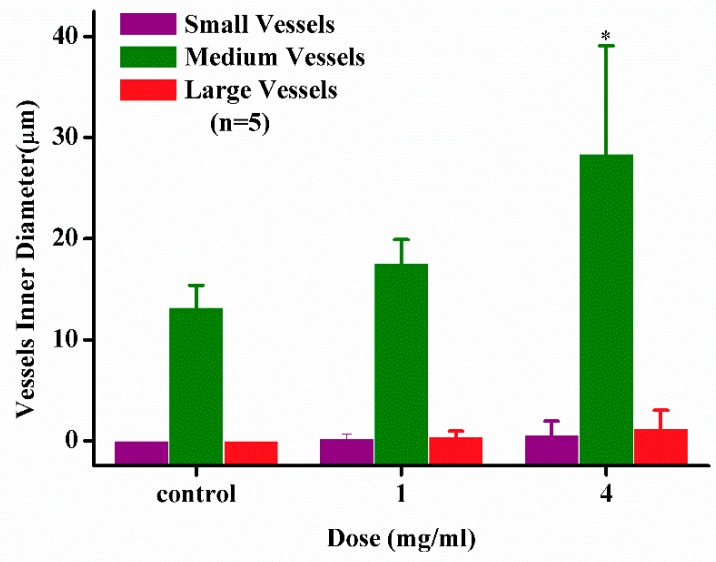
Effect of compound **8** on angiogenesis promotion. * *p* < 0.05; large vessels: Inner diameter > 100 µm; medium vessels: 10 µm < inner diameter < 100 µm; small vessels: Inner diameter < 10 µm.

Clinical practice has proved that angiogenic drugs could enhance the treatment efficacy of neuroprotection chemotherapy. Especially multi-effective neuroprotective agents present positive effects to neurosurgical patients. According to the references, baicalein can promote angiogenesis [37]. Analogously, the angiogenesis activities of compound **8** were evaluated by a CAM assay (Figure 4 and Figure 5). The model was established according to our previous work [36,37,38,39]. After implantation, the sponge is treated with a stimulator of blood vessel formation in the absence or presence of an angiogenesis inhibitor. Macroscopic observation shows that, the newly formed blood vessels grow radially around the gelatin sponge in the blank control group (Figure 4a). The high survival rate of embryos and normal growth of medium and large vessels (inner diameter > 50 µm) indicates successful modeling and low toxicity of compound **8** in vitro (Figure 5). Suppression of small vessels (inner diameter < 50 µm) were recognized as angiogenesis activity. We found compound **8** could dramatically promote small angiogenesis in a dose-dependent manner on CAM (Figure 4b,c), and with an increase of dosage, the degree of vascular promotion presents a good dose–effect relationship. Based on the above evidence, compound **8** might serve as an angiogenic drug that could enhance the treatment efficacy of neuroprotective chemotherapy.

## 3. Experimental Section

### 3.1. Chemistry

Reagents were bought from commercial suppliers and used without any further purification. 

Chemical shifts (*δ*) are given in ppm and coupling constants (*J*) in Hz. Melting points were measured at a rate of 5 °C/min using an X-5 micro melting point apparatus (Beijing Tektronix Department of Micron Technology Inc., Beijing, China). NMR spectra were recorded on a Bruker-500 spectrometer (Bruker, Dresden, Germany) with tetramethylsilane (TMS; TCI, Tokyo, Japan) as an internal standard; spectra are reported in *δ* (ppm). HR-MS were acquired using a Thermo Scientific TMLTQ Orbitrap XL hybrid FTMS instrument (Thermo Technologies, New York, NY, USA). Cellular morphologies were observed using an inverted fluorescence microscope (Olympus IX71, Tokyo, Japan).

#### General Procedure for the Preparation of Baicalein Amino Acid Derivatives **1**–**15**

The compound baicalein (1 equivalent (equiv.)) was dissolved in dry DCM (25 mL) and DMAP (0.5 equiv.), and the protected amino acid (1.2 equiv.) was added. After addition of EDCI (1.5 equiv.), the mixture was stirred at 25 °C for 12 h, protected by nitrogen. After completion of the reaction (as monitored by TLC), the solution was evaporated and washed with a saturated sodium carbonate solution (20 mL). The aqueous layer was extracted with DCM (25 mL), and the combined organic extracts were washed with brine (20 mL), dried over sodium sulfate, filtrated and evaporated. Purification was performed by flash chromatography.

The 5,7-dihydroxy-4-oxo-2-phenyl-4*H*-chromen-6-yl 2-{[(benzyloxy)carbonyl]amino}-3-methylbutanoate (compound **1**) was obtained as a white powder, with yield: 66.6%; m.p.: 199.7 °C, ${\left[\alpha \right]}_{D}^{20}$ = −41.03° (c 1, MeOH); ^1^H-NMR (500 MHz, Acetone-*d_6_*): *δ* (ppm) 13.19 (s, 1H, -OH), 9.57(s, 1H), 8.08 (d, 2H, *J =* 8.0 Hz), 7.60 (m, 3H), 7.37 (m, 5H), 6.83 (s, 1H), 6.74 (s, 1H), 5.14 (s, 2H), 4.50 (m, 1H), 2.90 (s, 1H), 1.16 (d, 6H, *J =* 6.5Hz); ^13^C-NMR (125 MHz, Acetone-*d_6_*): *δ* (ppm) 183.6, 170.4 (-COO-), 165.4, 158.2, 157.2, 155.9 (-COO-), 154.3, 137.9, 133.0, 132.3, 130.1, 129.4, 128.9, 128.9, 127.5, 106.0, 105.8, 95.4, 67.4, 60.9, 31.6, 30.4, 30.3, 30.1, 30.0, 29.8, 29.6, 29.5, 19.5, 18.3; HRMS (ESI) *m/z*: 502.1508 [M − H]^+^, calculatedd for C_28_H_25_NO_8_ = 502.1507.

The 6-[(2-{[(benzyloxy)carbonyl]amino-3,3-dimethylbutanoyl) oxy]-5-hydroxy-4-oxo-2-phenyl-4*H*-chromen-7-yl 2-{[(benzyloxy)carbonyl]amino-3,3-dimethylbutanoate (compound **2**) was obtained as white powder, with yield: 37.4%; m.p.: 195.8 °C, ${\left[\alpha \right]}_{D}^{20}$ = −27.18° (c 1, MeOH); ^1^H-NMR (500 MHz, Acetone-*d_6_*): *δ* (ppm) 13.34 (s, 1H, -OH), 8.14 (d, 2H, *J =* 7.0 Hz), 7.62 (m, 3H), 7.31 (m, 10H),7.16(s,1H) 6.98 (s, 2H),5.12(m,3H), 4.97(s,1H), 4.43(m,2H), 1.21 (d, 18H, *J =* 19.5 Hz); ^13^C-NMR (125 MHz, Acetone-*d_6_*): *δ* (ppm) 184.1, 169.8, 169.4, 166.3, 157.8 (-COO-), 154.4, 154.2, 149.5, 138.2, 138.0, 133.4, 131.9, 130.2, 130.1, 129.3, 129.3, 129.0, 128.9, 128.8, 127.7, 127.5, 109.9, 106.3, 102.9, 67.4, 67.2, 64.6, 64.2, 64.1, 35.4, 35.2, 30.4, 30.3, 30.1, 30.0, 29.8, 29.7, 29.5, 27.4, 27.2, 27.0; HRMS (ESI) *m/z*: 763.2867 [M − H]^+^, calculated for C_43_H_44_N_2_O_11_ = 763.2872. 

The 5,7-dihydroxy-4-oxo-2-phenyl-4*H*-chromen-6-yl 2-{[(benzyloxy) carbonyl] amino}-3,3-dimethylbutanoate (compound **3**) was obtained as a white powder, with yield: 30.2%; m.p.: 170.1 °C, ${\left[\alpha \right]}_{D}^{20}$ = −49.18° (c 1, MeOH); ^1^H-NMR (500 MHz, Acetone-*d_6_*): *δ* (ppm) 13.18 (s, 1H, -OH), 9.58(s,1H), 8.07 (d, 2H, *J =* 5.0 Hz), 7.60 (m, 3H), 7.37 (m, 5H), 7.01 (d, 1H, *J =* 5.0 Hz), 6.83 (s, 1H), 6.73 (s, 1H), 5.14 (s, 2H), 4.54 (m, 1H), 2.95 (s, 1H), 1.42 (m, 1H), 1.16(d, 4H, *J =* 7.0 Hz), 1.00 (m, 4H).^13^C-NMR (125 MHz, Acetone-*d_6_*): *δ* (ppm) 183.6, 170.3 (-COO-), 165.3, 158.2, 157.2, 155.8 (-COO-), 154.2, 137.9, 132.9, 132.2, 130.1, 129.3, 128.9, 128.9, 127.4, 123.2, 106.0, 105.9, 105.8, 95.5, 95.4, 67.4, 60.1, 60.0, 38.2, 30.4, 30.2, 30.1, 29.9, 29.8, 29.6, 29.5, 25.8, 15.8, 12.0; HRMS (ESI) *m/z*: 516.1680 [M − H]^+^, calculated for C_29_H_27_NO_8_ = 516.1644.

The 1-benzyl 5,7-dihydroxy-4-oxo-2-phenyl-4*H*-chromen-6-yl 2-{[(benzyloxy)carbonyl]amino} butanedioate (compound **4**) was obtained as a white powder, with yield: 45.2%; m.p.: 157.8 °C, ${\left[\alpha \right]}_{D}^{20}$ = −7.84° ( c 1, MeOH); ^1^H-NMR (500 MHz, Acetone-*d_6_*): *δ* (ppm) 13.18 (s, 1H, -OH),9.86(s,1H), 8.07 (d,2H, *J =* 6.5Hz), 7.59 (m, 3H), 7.35 (m, 10H), 6.83 (s, 1H), 6.74 (s, 1H), 5.22 (s, 3H), 5.13 (s, 3H).^13^C-NMR (125 MHz, Acetone-*d_6_*): *δ* (ppm) 183.6, 168.8, 165.3, 157.1, 155.7 (-COO-), 154.1, 138.0, 1367.0, 133.3, 133.0, 132.2, 130.2, 130.1, 129.4, 129.4, 129.3, 129.1, 129.0, 128.8, 128.8, 128.7, 128.7, 127.7, 127.4, 105.9, 95.3, 67.9, 67.1, 51.9, 36.9, 30.4, 30.2, 30.1, 29.9, 29.8, 29.6, 29.5; HRMS (ESI) *m/z*: 608.1581 [M − H]^+^, calculated for C_34_H_27_NO_10_ = 608.1562.

The 5,6-dihydroxy-4-oxo-2-phenyl-4*H*-chromen-7-yl 2{[(tert-butoxy) carbonyl](methyl)amino}acetate (compound **5**) was obtained as a white powder, with yield: 40.6 %; m.p.: 218.0 °C, ${\left[\alpha \right]}_{D}^{20}$ = −7.28° (c 1, MeOH); ^1^H-NMR (500 MHz, CDCl_3_): *δ* (ppm) 12.98 (s, 1H, -OH of baicalin), 8.55 (s, 1H, C6-OH), 7.87 (d, 2H, *J* = 7.0 Hz), 7.52 (m, 3H), 6.64 (s, 2H, *J* = 16.5Hz), 4.13 (s, 2H), 3.12 (s, 3H), 1.51 (s, 9H, 3 × -CH_3_ of Boc).^13^C-NMR (125 MHz, CDCl_3_): *δ* (ppm) 182.8, 167.1 (-COO-), 164.5, 158.2 (-COO-), 156.0, 155.3, 153.3, 132.1, 131.4, 129.2, 126.5, 122.0, 105.4, 105.3, 95.2, 82.4, 52.1, 37.33, 31.1, 29.8, 28.5; HRMS (ESI) *m/z:* 440.1355 [M − H]^+^, calculated for C_23_H_23_NO_8_ = 440.1351.

The 5,7-dihydroxy-4-oxo-2-phenyl-4*H*-chromen-6-yl 2-{[(tert-butoxy)carbonyl]amino}-3-phenylpropanoate (compound **6**) was obtained as a white powder, yield: 42.0%; m.p.: 155.2 °C, ${\left[\alpha \right]}_{D}^{20}$ = −6.02° (c 1, MeOH); ^1^H-NMR (500 MHz, Acetone-*d_6_*): *δ* (ppm) 13.18 (s, 1H, -OH), 9.69(s,1H), 8.00 (d, 3H, *J =* 7.0Hz), 7.59 (d, 5H, *J =* 7.5Hz), 7.33 (m, 3H), 6.82 (s, 1H), 6.74 (s, 1H), 4.70 (m, 1H), 1.39 (s, 9H, 3 × -CH_3_ of Boc); ^13^C-NMR (125 MHz, Acetone-*d_6_*): *δ* (ppm) 183.6, 170.4 (-COO-), 165.2, 157.42, 157.23, 155.8 (-COO-), 154.2, 138.1, 132.9, 132.2, 130.4, 130.3, 130.1, 130.1, 129.3, 127.6, 127.4, 123.3, 105.9, 105.7, 95.4, 80.5, 56.3, 56.2, 38.1, 30.4, 30.2, 30.1, 29.9, 29.8, 29.6, 29.4, 28.7, 28.7, 28.6, 28.3; HRMS (ESI) *m/z*: 550.1505 [M − H]^+^, calculated for C_32_H_25_NO_8_ = 550.1507.

The 5,6-dihydroxy-4-oxo-2-phenyl-4*H*-chromen-7-yl 2-{[(benzyloxy)carbonyl]amino}-3-methyl-pentanoate (compound **7**) was obtained as a white powder, with yield: 43.1%; m.p.: 155.7 °C, ${\left[\alpha \right]}_{D}^{20}$ = −9.84° (c 1, MeOH); ^1^H-NMR (500 MHz, CDCl_3_): *δ* (ppm) 12.84 (s, 1H, -OH), 7.80 (d, 2H, *J =* 8.5 Hz), 7.46 (m, 4H), 6.98 (s, 1H), 6.68 (d, 1H, *J =* 7.0 Hz), 5.59 (s, 1H), 5.18 (s, 1H), 4.50 (s, 1H), 4.43 (s, 1H), 2.09 (s, 2H), 1.45 (s, 9H); ^13^C-NMR (125 MHz, CDCl_3_): *δ* (ppm) 182.8, 169.32, 165.0, 156.2 (-COO-), 155.6, 153.4, 153.2, 148.4, 132.4, 130.6, 130.6, 129.2, 126.4, 126.4, 125.9, 109.3, 105.6,101.4, 80.6, 80.0, 80.0, 59.1, 58.1, 37.8, 37.0, 29.4, 29.3, 28.5, 28.4, 26.9, 25.4, 24.9, 22.8, 19.3, 19.2, 15.7, 15.6 11.8, 11.4; HRMS (ESI) *m/z*: 516.1663 [M − H]^+^, calculated for C_29_H_27_NO_8_ = 516.1664.

The 5,7-dihydroxy-4-oxo-2-phenyl-4*H*-chromen-6-yl 2-{[(benzyloxy)carbonyl]amino}-3-phenyl-proppanpate (compound **8**) was obtained as a white powder, with yield: 36.6%; m.p.: 161.3 °C, ${\left[\alpha \right]}_{D}^{20}$ = −3.85° (c 1, MeOH); ^1^H-NMR (500 MHz, Acetone-*d_6_*): *δ* (ppm) 13.18 (s, 1H, -OH), 9.69(s, 1H), 8.06 (d, 3H, *J =* 7.0Hz), 7.59 (d, 4H, *J =* 7.5Hz), 7.33 (m, 3H), 6.82 (s, 1H), 6.74 (s, 1H), 5.08 (s, 2H), 4.70 (m, 1H), 1.39 (s, 9H); ^13^C-NMR (125 MHz, Acetone-*d_6_*):*δ* (ppm) 183.6, 170.2 (-COO-), 165.3, 157.6, 157.2, 155.8 (-COO-), 154.2, 138.1, 137.9, 133.0, 132.2, 130.4, 130.2, 130.1, 129.4, 129.3, 128.8, 128.7, 127.7, 127.4, 123.4, 106.0, 105.9, 105.8, 95.5, 95.4, 67.2, 56.6, 56.5, 38.3, 30.4, 30.2, 30.1, 29.9, 29.8, 29.6, 29.5, 27.6; HRMS (ESI) *m/z*: 516.1665 [M − H]^+^, calculated for C_29_H_27_NO_8_ = 516.1644.

The 1-benzyl 5,7-dihydroxy-4-oxo-2-phenyl-4*H*-chromen-6-yl 3-{[(benzyloxy) carbonyl] amino} butanedioate (compound **9**) was obtained as a white powder, with yield: 37.4; m.p.: 100.7 °C, ${\left[\alpha \right]}_{D}^{20}$ = −3.48° (c 1, MeOH); ^1^H-NMR (500 MHz, Acetone): *δ* (ppm) 13.15 (s, 1H, -OH), 9.69(s, 1H), 8.07 (d, 3H, *J =* 8.0Hz), 7.36 (m, 15H), 6.82 (s, 1H), 6.72 (s, 1H), 5.19 (s, 3H), 5.15 (s, 3H); ^13^C-NMR (125 MHz, Acetone-*d_6_*): *δ* (ppm) 185.5, 172.9, 171.6, 167.3, 167.2, 159.4, 159.0, 157.8, 156.1, 155.8 (-COO-), 139.8, 139.1, 134.9, 134.2, 132.1, 131.4, 131.3, 131.3, 131.0, 131.0, 130.9, 130.8, 130.8, 129.4, 129.2, 125.2, 108.0, 107.9, 107.8, 97.5, 97.4, 69.4, 69.3, 69.0, 53.8, 53.7, 53.5, 39.5, 39.3, 32.4, 32.2, 32.1, 31.9, 31.8, 31.6, 31.4; HRMS (ESI) *m/z*: 608.1563 [M − H]^+^, calculated for C_34_H_27_NO_10_ = 608.1562.

The 5,6-dihydroxy-4-oxo-2-phenyl-chroman-7-yl 2-{[(tert-butoxy)carbonyl]amino}-3-methyl-pentanoate (compound **10**) was obtained as white powder, with yield: 56.3%; m.p.: 165.2 °C, ${\left[\alpha \right]}_{D}^{20}$ = −20° (c 1, MeOH); ^1^H-NMR (500 MHz, CDCl_3_): *δ* (ppm) 7.87 (d, 2H, *J =* 7.0Hz), 7.52 (m, 4H), 6.65 (s, 1H, C3-H), 6.62 (s, 1H, C8-H), 5.17 (s, 1H), 4.18 (s, 1H),1.48 (s, 9H, 3 × -CH_3_ of Boc), 1.20 (d, 3H, *J =* 6.5Hz), 1.02 (s, 3H); ^13^C-NMR (125 MHz, CDCl_3_): *δ* (ppm) 182.8, 170.6(-COO-), 164.5, 157.5 (-COO-), 155.99, 156.0, 155.2, 153.2, 132.0, 131.4, 129.2, 126.5, 122.0, 105.5, 105.4, 95.1, 82.1, 77.4, 77.2, 76.9, 59.4, 36.5, 28.5, 25.6, 15.4, 11.3;HRMS (ESI) *m/z*: 482.1834 [M − H]^+^, calculated for C_26_H_29_NO_8_ = 482.182.

The 1-tert-butyl 2-(5,7-dihydroxy-4-oxo-2-phenyl-chroman-6-yl) pyrrolidine-1,2-dicarboxylate (compound **11**) was obtained as a white powder, with yield: 23.5%; m.p.: 109.8 °C, ${\left[\alpha \right]}_{D}^{20}$ = −29.09° (c 1, MeOH); ^1^H-NMR (500 MHz, CDCl_3_): *δ* (ppm) 12.91 (s, 1H, -OH), 8.83(s,1H), 7.90 (d, 2H, *J* = 7.0Hz), 7.54 (m, 3H), 6.67 (1H, s, C3-H), 6.64 (1H, s, C8-H), 1.54 (s, 9H, 3 × -CH_3_ of Boc); ^13^C-NMR (125 MHz, CDCl_3_): *δ* (ppm) 182.8, 170.0(-COO-), 164.4, 156.5, 156.2, 155.3, 153.3, 132.0, 131.5, 129.2, 126.5, 121.9, 105.5, 105.3, 95.1, 82.0, 59.1, 47.3, 30.3, 29.8, 28.6, 28.5, 28.2; HRMS (ESI) *m/z*: 466.1511 [M − H]^+^, calculated for C_25_H_25_NO_8_ = 446.1507.

The 5,6-dihydroxy-4-oxo-2-phenyl-4*H*-chromen-7-yl 2,6-di{[(benzyloxy)carbonyl] amino} hexanoate (compound **12**) was obtained as a white powder, with yield: 7.8%; m.p.:144.9 °C, ${\left[\alpha \right]}_{D}^{20}$ = −3.20° (c 1, MeOH); ^1^H-NMR (500 MHz, CDCl_3_):*δ* (ppm) 12.93 (s, 1H), 8.43(s,1H), 7.86 (s, 2H), 7.52 (m, 3H), 7.28 (m, 13H), 6.63 (s, 1H), 6.61 (s, 1H), 5.12 (m, 8H); ^13^C-NMR (125 MHz, CDCl_3_): *δ* (ppm) 182.8, 169.9, 164.5, 158.1, 157.3 (-COO-), 155.8, 155.2, 153.1, 136.6, 135.7, 132.1, 131.3, 131.1, 129.2, 129.0, 128.7, 128.6, 128.5, 128.4, 128.2, 128.2, 126.5, 122.0, 105.4, 105.4, 95.1, 68.0, 67.0, 65.7, 54.9, 39.8, 30.7, 30.1, 29.6, 27.1, 21.6, 19.3, 13.9; HRMS (ESI) *m/z*: 665.2134 [M − H]^+^, calculated for C_37_H_34_N_2_O_10_ = 665.2141.

The 7-[(2-{[(tert-butoxy)carbonyl](methyl)amino}acetyl)oxy]-5-hydroxy-4-oxo-2-phenyl-4*H*-chromen-6-yl 2-{[(tert-butoxy)carbonyl](methyl)amino}acetate (compound **13**) was obtained as a white powder, with yield: 46.9%; m.p.:205.1 °C, ${\left[\alpha \right]}_{D}^{20}$ =−3.92° (c 1, MeOH); ^1^H-NMR (500 MHz, CDCl_3_):*δ* (ppm) 12.97 (s, 1H), 7.87 (d, 2H, *J =* 7.0Hz), 7.52 (m, 4H), 6.65 (s, 1H, C3-H), 6.61 (s, 1H, C8-H), 4.14 (s, 2H), 3.12 (s, 3H), 1.51 (s, 9H, 3 × -CH_3_ of Boc); ^13^C-NMR (125 MHz, CDCl_3_): *δ* (ppm) 182.8, 167.1 (-COO-), 164.5, 158.2 (COO-), 156.0, 155.3, 153.3, 132.1, 131.4, 129.2, 126.5, 122.0, 105.4, 105.3, 95.2, 82.4, 52.1, 37.33, 31.1, 29.8, 28.5; HRMS (ESI) *m/z*: 611.2240 [M − H]^+^, calculated for C_31_H_36_N_2_O_11_ = 611.2246.

The 5,7-dihydroxy-4-oxo-2-phenyl-4*H*-chromen-6-yl 2-{[(tert-butoxy) carbonyl] amino} propanoate (compound **14**) was obtained as a white powder, with yield: 36.7%; m.p.: 196.7 °C, ${\left[\alpha \right]}_{D}^{20}$ = −29.91° (c 1, MeOH); ^1^H-NMR (400 MHz, CDCl_3_): *δ* (ppm) 13.13 (s, 1H, -OH), 9.56 (s, 1H, C6-OH), 8.08 (d, 3H, *J =* 7.0 Hz), 7.60 (d, 3H, *J =* 6.9 Hz), 6.82 (s, 1H), 6.72 (s, 1H), 4.52 (m, 1H), 1.59 (d, 3H, *J =* 7.1 Hz), 1.46 (s, 9H); ^13^C-NMR (101 MHz, Dimethyl sulfoxide (DMSO)-*d_6_*): *δ* (ppm) 205.4, 182.6, 182.5, 170.5 (-COO-), 164.4, 164.3, 156.7, 156.2, 154.9 153.2, 153.0, 132.0, 131.3, 129.1, 126.5, 105.3, 104.4, 94.4, 79.6, 49.6, 28.3, 27.7, 16.9; HRMS (ESI) *m/z*: 440.1355 [M − H]^+^, calculated for C_23_H_23_NO_8_ = 440.1351. 

The 5,6-dihydroxy-4-oxo-2-phenyl-4*H*-chromen-7-yl 2-{[(tert-butoxy) carbonyl] amino}-4-methyl-pentanoate (compound **15**) was obtained as a white powder, with yield: 44.6%; m.p.: 199.3 °C, ${\left[\alpha \right]}_{D}^{20}$ = −17.54° (c 1, MeOH); ^1^H-NMR (500 MHz, CDCl_3_): *δ* (ppm) 12.89 (s, 1H),8.37(s,1H), 7.87 (d, 2H, *J =* 7.0Hz), 7.52 (m, 3H), 6.65 (s, 1H, C3-H), 6.62 (s, 1H, C8-H), 1.48 (s, 9H, 3 × -CH_3_ of Boc), 1.05 (s, 3H), 1.01 (s, 3H); ^13^C-NMR (125 MHz, CDCl_3_): *δ* (ppm) 182.8, 170.6 (-COO-), 164.5, 157.5 (-COO-), 156.0, 155.2, 153.2, 132.0, 131.4, 129.2, 126.5, 122.0, 105.5, 105.4, 95.1, 82.1, 77.4, 77.2, 76.9, 53.4, 40.0, 29.8, 28.5, 24.9, 22.7, 22.4; HRMS (ESI) *m/z*: 482.1822 [M − H]^+^, calculated for C_26_H_29_NO_8_ = 482.182.

### 3.2. Bio-Evaluation Methods

#### 3.2.1. Cell Culture

The SH-SY5Y (liver neurons) and H9C2 (human cardiac myocytes) cell lines were obtained from the Chinese Academy of Medical Sciences and Peking Union Medical College. All of the cell lines were grown in dulbecco’s modified eagle medium (DMEM) with 10% (*v/v*) fetal bovine serum (FBS) and 1% (*v/v*) penicillin/streptomycin (Thermo Technologies, New York, NY, USA), under a humidified atmosphere containing 5% CO_2_ at 37 °C. The baicalein derivatives were dissolved in DMSO (Sigma, St. Louis, MO, USA) and added at various concentrations to the cell culture.

#### 3.2.2. Protective Effect on Injured SH-SY5Y Cells

The protective effect of these compounds was evaluated on SH-SY5Y and H9C2 cell lines via the MTT method. The cells growing in the logarithmic phase were cultured in 96-well plates at a density 3 × 10^3^ cells/well and incubated for 24 h. Then each well was treated with the required concentrations (50 µM concentrations, 25 µM concentrations, 12.5µM concentrations, 6.25 µM concentrations, and 3.125 µM concentrations) of baicalein derivatives, and incubated 36 h at 37 °C with 5% CO_2_. After that, the cells were induced by H_2_O_2_ (final concentration, 200 mM) for 12 h. Control-differentiated cells were injected with new growth media at equal amounts. Before 20 mL MTT solution (5 mg/mL) was added to each well, the cell culture fluid was discarded, and 100 mL fresh cell culture fluid was added. The plate was incubated for a further 4 h at 37 °C. The cell supernatant medium was removed carefully, without disturbing the attached cells, and formazan crystals were solubilized by adding 150 μL DMSO into each well. The absorbance was measured at 490 nm with a plate reader (BIORAD 550 spectrophotometer, Bio-rad Life Science Development Ltd., Beijing, China). H_2_O_2_ was dissolved in DMEM medium. Wells without drugs were used as blanks. The EC_50_ values were defined as the concentrations that causes the maximum effect of 50%; IC_50_ values were defined as the concentration of compound which gives 50% growth inhibition, and were calculated using the GraphPad Prism 5. The proliferation rates of damaged SH-SY5Y cells were calculated in the following formula (1):

[OD_490_ (compound) − OD_490_ (CoCl_2_)]/[OD_490_ (NGF) − OD_490_ (CoCl_2_)] × 100% (1)

The EC_50_ values were using the equation

−pEC_50_ = log *C_max_* − log 2 × (Σ*P* − 0.75 + 0.25*P_max_* + 0.25*P_min_*) (2)

where *C_max_* is the maximum concentration, Σ*P* is the sum of proliferation rates, *P_max_* is the maximum value of the proliferation rate, and *P_min_* is the minimum value of the proliferation rate. Cell viability was expressed as a percentage of the control. 

The inhibition rate was calculated in the following equation:

% inhibition = 1 − (Sample group OD/Control group OD) × 100%(3)

#### 3.2.3. Giemsa Staining

SH-SY5Y cells in the logarithmic growth phase were seeded in 12-well plates (1.2 × 10^4^ cells/well). After incubation for 24 h at 37 °C with 5% CO_2_, various concentrations (0, 2, 4, or 6 μM concentrations) of compound **8** were added to the cultures, and the plate was incubated for a further 72 h. Then, we discarded the cell culture fluid, washed twice with phosphate-buffered saline (PBS), and fixed the cells with cold methanol. The SH-SY5Y cells were stained with 6% Giemsa solution for 5 min, washed with water, and dried. The cell morphological changes were observed under an inverted microscope.

#### 3.2.4. DAPI Staining

SH-SY5Y cells in logarithmic growth phase were seeded in 12-well plates (2.4 × 10^4^ cells/well). After incubation for 24 h at 37 °C in a humidified atmosphere with 5% CO_2_, various concentrations of compound **8** was added to the cultures, and the plate was incubated for further 72 h. After discarding the cell culture medium, washing twice with PBS, and fixing with 4% paraformaldehyde for 10 min, 4′,6-diamidino-2-phenylindole (DAPI, 1 mg/mL, Molecular Probes/Invitrogen Life Technologies, Carlsbad, CA, USA), staining was performed for 5 min in the dark, and nuclear fragments were observed under fluorescence microscope.

#### 3.2.5. Angiogenesis Assay

Fertilized White Leghorn chicken eggs (50–65 g), provided by the Chinese Academy of Agricultural Sciences, were placed in an incubator as soon as embryogenesis started, and were kept under constant humidity of 65% at 37 °C. Here, we present a method for the angiogenesis in the chick embryo chorioallantoic membrane (CAM), based on the implantation of a gelatin sponge on the top of the growing CAM on day 7 of development. On day 7, under sterile conditions, a square window was opened on the shell, and physiological saline (0.1 mL) was injected in to detach the shell membrane. Gelatin sponges were implanted, respectively. Moreover, 1 mm sterilized gelatin sponges carrying the compound **8** dissolved in saline at 1 and 4 mg/mL were implanted on the smaller vessel part of the CAM. The window was sealed with sterile adhesive, and the eggs were returned to the incubator for a further 48 h. Then, the tapes were removed, and the entire CAM was detached after tissue fixation with methanol/acetone (1:1, *v/v*). We use computer-assisted tracking of images to obtain absolute values for the number of microvessels. Quantitative evaluation of the angiogenic response, expressed as microvessel density, can be obtained by applying a morphometric method of “point counting” on histological CAM sections. Data was analyzed using a *t*-test of the statistics analysis system; the values were expressed as mean ± SD of six observations, and *p* < 0.05 was considered significant.

### 3.3. Statistical Analysis

All data were expressed as the mean ± standard deviation (SD) of three replications. The statistical analysis was performed by SPSS software (Version 20.0) to analyze the variance. One-way analysis of variance (ANOVA) was followed by the least significant difference (LSD) post-hoc test for multiple comparisons. A *p*-value of less than 0.05 was considered significant.

## 4. Conclusions

In this study, 15 novel baicalein derivatives have been designed and synthesized by attaching different amino acids. All of them were characterized by ^1^H-NMR, ^13^C-NMR, and HRMS (Figure S6), and tested for biological activities against SH-SY5Y and H9C2 cells, as well as a CAM model. The results indicate that most of the synthesized baicalein amino acid derivatives were more potent than the positive drugs baicalein, and were equivalent to the positive drug edaravone. Among them, compound **8** exhibited significant neuroprotective activity on the SH-SY5Y cell line (EC_50_ = 4.31 μM) and lower toxicity on the H9C2 cell line. The detection of apoptosis and cell cycle analysis indicates that compound **8** could protect injured SH-SY5Y cells from nuclear fragmentation and effectively protect nerves. Furthermore, the CAM test revealed that compound **8** also had the ability to promote angiogenesis effectively. The experimental results show that free radical formed by dehydrogenation of 6-OH is most stable, and the hydrogen can be easily replaced. Cbz-protected baicalein amino acid derivatives showed better neuroprotective activity than boc-protected baicalein amino acid derivatives. Our current findings provided a meaningful reference for further research on vascular protection mechanisms of baicalein derivatives.

## Figures and Tables

**Table 1:** The structures of baicalein derivatives **1**–**15**.

Compound	Structure	Compound	Structure
**1**	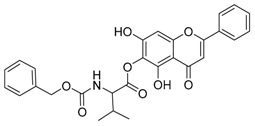	**2**	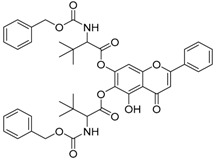
**3**	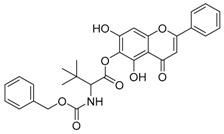	**4**	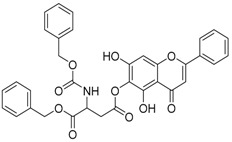
**5**	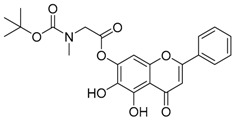	**6**	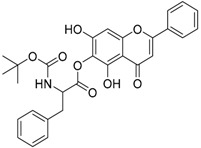
**7**	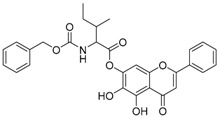	**8**	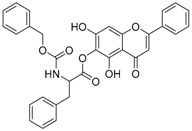
**9**	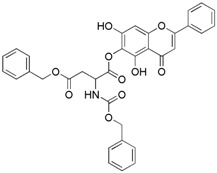	**10**	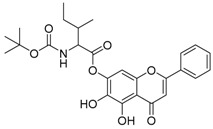
**11**	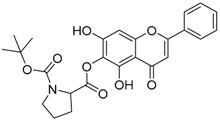	**12**	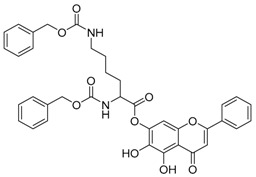
**13**	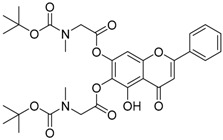	**14**	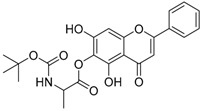
**15**	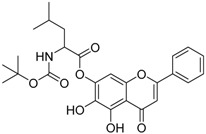		

**Table 2:** The 50% effective concentration (EC_50_) of baicalein derivatives for protecting injured SH-SY5Y cells.

Compound	Proliferation Rate (%)					EC_50_ (µM)
	50 µM	25 µM	12.5 µM	6.25 µM	3.125 µM	
**1**	54.92	67.35	81.81	84.35	55.99	6.07
**2**	94.24	106.84	81.03	48.22	45.07	4.79
**3**	−0.18	0.15	8.10	14.82	15.27	62.36
**4**	54.80	70.23	77.70	94.11	59.08	5.51
**5**	60.70	78.11	76.37	37.56	28.36	9.96
**6**	16.74	21.58	24.75	19.99	8.61	42.04
**7**	72.17	79.76	79.76	69.72	63.62	5.24
**8**	94.03	87.31	83.08	75.12	52.49	4.31
**9**	84.15	101.00	79.58	98.20	28.13	4.47
**10**	63.22	74.55	79.27	77.21	75.23	5.07
**11**	64.80	74.48	78.16	86.25	47.94	5.82
**12**	7.34	20.81	7.83	2.35	1.10	61.60
**13**	65.86	72.99	91.54	92.10	40.12	5.42
**14**	0.10	4.09	7.09	3.34	0.35	74.86
**15**	22.31	41.27	42.51	42.76	17.81	23.85
Baicalein	11.69	28.36	29.85	35.07	54.73	24.77
Edaravone	60.70	69.90	76.12	85.82	61.44	5.62

**Table 3:** The 50% inhibition concentration (IC_50_) of the baicalein derivatives for H9C2 cells.

Compound	Survival Rate (%)					IC_50_ (µM)
	100 µM	50 µM	25 µM	12.5 µM	6.25 µM	
**1**	204.12	215.52	231.16	260.35	269.76	>100.00
**2**	107.88	112.46	133.06	149.84	213.19	>100.00
**3**	104.18	132.42	134.97	137.27	144.06	>100.00
**4**	125.68	130.55	140.46	140.39	155.60	>100.00
**5**	76.93	95.13	105.32	115.71	115.66	>100.00
**6**	121.68	137.47	153.61	154.46	157.32	>100.000
**7**	123.82	138.56	144.70	146.75	152.03	>100.00
**8**	167.58	168.17	169.08	182.13	195.31	>100.00
**9**	107.43	114.84	129.50	133.10	152.78	>100.00
**10**	141.70	152.13	169.33	179.84	194.73	>100.00
**11**	45.75	60.67	61.98	67.32	70.98	95.21
**12**	183.88	187.47	200.19	206.64	216.11	>100.00
**13**	123.43	132.48	151.84	166.23	215.46	>100.00
**14**	97.10	129.44	137.29	166.30	184.85	>100.00
**15**	159.21	172.74	177.39	193.04	196.35	>100.00
Baicalein	168.20	169.76	178.56	198.36	212.09	>100.00

## Supplementary Materials

The following are available online. Figure S6: ^1^H-NMR, ^13^C-NMR, and HR-MS spectra of compound **1**–**15**.

## References

[1] Tejada S., Setzer W.N., Daglia M., Nabavi S.F., Sureda A., Braidy N., Gortzi O., Nabavi S.M. (2016). Neuroprotective effects of Ellagitannins: A brief review. Curr. Drug Targets.

[2] Losada-Barreiro S., Bravo-Dã-Az C. (2017). Free radicals and polyphenols: The redox chemistry of neurodegenerative diseases. Eur. J. Med. Chem..

[3] Hsieh H.L., Yang C.M. (2013). Role of redox signaling in neuroinflammation and neurodegenerative disease. BioMed. Res. Int..

[4] Niloufar A., Fariba K. (2013). Natural Products as Promising Drug Candidates for the Treatment of Alzheimer’s Disease: Molecular Mechanism Aspect. Curr. Neuropharmacol..

[5] Sowndhararajan K., Kim S. (2017). Neuroprotective and Cognitive Enhancement Potentials of Angelica gigas Nakai Root: A Review. Sci. Pharm..

[6] Hirsch E.C., Vyas S., Hunot S. (2012). Neuroinflammation in Parkinson’s disease. Parkinsonism Relat. Disord..

[7] Chen W.W., Zhang X., Huang W.J. (2016). Role of neuroinflammation in neurodegenerative diseases. Mol. Med. Rep..

[8] Desai A.K., Grossberg G.T. (2005). Diagnosis and treatment of Alzheimer’s disease. Neurology.

[9] Doody R.S., Ferris S.H., Salloway S., Sun Y., Goldman R., Watkins W.E., Xu Y., Murthy A.K. (2009). Donepezil treatment of patients with MCI: A 48-week randomized, placebo-controlled trial. Neurology.

[10] Mizuno Y. (2014). Recent research progress in and future perspective on treatment of Parkinson’s disease. Integr. Med. Int..

[11] Okun M.S. (2014). Deep-brain stimulation—Entering the era of human neural-network modulation. N. Engl. J. Med..

[12] Fang J., Pang X., Yan R., Lian W., Li C., Wang Q., Du G.H. (2016). Discovery of neuroprotective compounds by machine learning approaches. RSC Adv..

[13] Fang J., Liu C., Wang Q., Lin P., Cheng F. (2017). In silico polypharmacology of natural products. Brief. Bioinform..

[14] Chen X., Pan W. (2014). The treatment strategies for neurodegenerative diseases by integrative medicine. Integr. Med. Int..

[15] Fang J., Li Y., Liu R., Pang W., Li C., Yang R., He Y., Lian W., Liu A.L., Du G.H. (2015). Discovery of multitarget-directed ligands against Alzheimer’s disease through systematic prediction of chemical–protein interactions. J. Chem. Inf. Model..

[16] Pak M.E., Kim Y.R., Kim H.N., Ahn S.M., Shin H.K., Baek J.U., Choi B.T. (2016). Studies on medicinal herbs for cognitive enhancement based on the text mining of Dongeuibogam and preliminary evaluation of its effects. J. Ethnopharmacol..

[17] Grossberg G.T. (2003). Diagnosis and treatment of Alzheimer’s disease. J. Clin. Psychiatry.

[18] Crane P.K., Doody R.S. (2009). Donepezil treatment of patients with MCI: A 48-week randomized, placebo-controlled trial. Neurology.

[19] Xiao J.R., Do C.W., To C.H. (2014). Potential therapeutic effects of baicalein, baicalin and wogonin in ocular disorders. J. Ocul. Pharmacol. Ther..

[20] de Oliveira M.R., Nabavi S.F., Habtemariam S., Orhan I.E., Daglia M., Nabavi S.M. (2015). The effects of baicalein and baicalin on mitochondrial function and dynamics: A review. Pharmacol. Res..

[21] Gao Y., Snyder S.A., Smith J.N., Chen Y.C. (2016). Anticancer properties of baicalein: A review. Med. Chem. Res..

[22] Li Y., Zhao J., Hölscher C. (2017). Therapeutic Potential of baicalein in Alzheimer’s disease and Parkinson’s disease. CNS Drugs.

[23] Muto T., Motozuka M., Nakano Y., Tatsumi F. (1998). The chemical structure of new substance as the metabolite of baicalin and time profiles for the plasma concentration after oral administration of Sho-saiko-to in human. J. Pharm. Soc. Jpn..

[24] Zhang L., Lin G., Zuo Z. (2004). High-performance liquid chromatographic method for simultaneous determination of baicalein and baicalein 7-glucuronide in rat plasma. J. Pharm. Biomed. Anal..

[25] Taiming L., Xuehua J. (2006). Investigation of the absorption mechanisms of baicalin and baicalein in rats. J. Pharm. Sci..

[26] Lai M.Y., Hsiu S.L., Tsai S.Y., Hou Y.C., Chao P.D. (2010). Comparison of metabolic pharmacokinetics of baicalin and baicalein in rats. J. Pharm. Pharmacol..

[27] Smolders I., Khan G.M., Lindekens H., Prikken S., Marvin C.A., Manil J., Ebinger G., Michotte Y. (1997). Effectiveness of vigabatrin against focally evoked pilocarpine-induced seizures and concomitant changes in extracellular hippocampal and cerebellar glutamate, gamma-aminobutyric acid and dopamine levels, a microdialysis-electrocorticography study in freely moving rats. J. Pharmacol. Exp. Ther..

[28] Heales S.J., Bolaños J.P., Stewart V.C., Brookes P.S., Land J.M., Clark J.B. (1999). Nitric oxide, mitochondria and neurological disease. Biochim. Et Biophys. Acta (BBA)-Bioenerg..

[29] Frugier G., Coussen F., Giraud M.F., Odessa M.F., Emerit M.B., Boué-Grabot E., Garret M. (2007). A gamma 2(R43Q) mutation, linked to epilepsy in humans, alters GABAA receptor assembly and modifies subunit composition on the cell surface. J. Biol. Chem..

[30] Ding R., Asada H., Obata K. (1998). Changes in extracellular glutamate and GABA levels in the hippocampal CA3 and CA1 areas and the induction of glutamic acid decarboxylase-67 in dentate granule cells of rats treated with kainic acid. Brain Res..

[31] Xiudao S., Yunmei L., Jin M., Jun H., Xing Z., Xiaoyong L., Guorong J., Lurong Z. (2015). Synthesis of novel amino acid derivatives containing chrysin as anti-tumor agents against human gastric carcinoma MGC-803 cells. Med. Chem. Res..

[32] Brady S.F., Pawluczyk J.M., Lumma P.K., Feng D.M., Wai J.M., Jones R., Oliff A. (2002). Design and synthesis of a pro-drug of vinblastine targeted at treatment of prostate cancer with enhanced efficacy and reduced systemic toxicity. J. Med. Chem..

[33] DeFeo-Jones D., Brady S.F., Feng D.M., Wong B.K., Bolyar T., Haskell K., Kiefer D.M., Leander K., McAvoy E., Lumma P. (2002). A prostate-specific antigen (PSA)-activated vinblastine prodrug selectively kills PSA-secreting cells in vivo. Mol. Cancer Ther..

[34] Fang K., Zhang X.H., Han Y.T., Wu G.R., Cai D.S., Xue N.N., Guo W.B., Yang Y.Q., Chen M., Zhang X.Y. (2018). Design, Synthesis, and Cytotoxic Analysis of Novel Hederagenin–Pyrazine Derivatives Based on Partial Least Squares Discriminant Analysis. Int. J. Mol. Sci..

[35] Biegon A. (2004). Cannabinoids as Neuroprotective Agents in Traumatic Brain Injury. Curr. Pharm. Des..

[36] Lee D.Y., Kim S.K., Kim Y.S., Son D.H., Nam J.H., Kim I.S., Park R.W., Kim S.Y., Byun Y. (2007). Suppression of angiogenesis and tumor growth by orally active deoxycholic acid-heparin conjugate. J. Control. Release..

[37] Li H.Q., Wu X.Z., Bai D., Yang Y.N., Lei H.M. (2011). Screening active fraction of compound Sanhuang capsules for inhibition of angiogenesis in tumor. Chin. J. Exp. Tradit. Med. Formulae.

[38] Wang P., She G., Yang Y., Li Q., Zhang H., Liu J., Lei H. (2012). Synthesis and Biological Evaluation of New Ligustrazine Derivatives as Anti-Tumor Agents. Molecules.

[39] Li B., Yan W., Zhang C., Zhang Y., Liang M., Chu F., Lei H. (2015). New Synthesis Method for Sultone Derivatives: Synthesis, Crystal Structure and Biological Evaluation of S-CA. Molecules.

