# Supplementary material for: Synthesis of Novel Baicalein Amino Acid Derivatives and Biological Evaluation as Neuroprotective Agents
Source: Molecules. 2019 Oct 9;24(20):3647. doi: 10.3390/molecules24203647 (PMC6832219; doi:10.3390/molecules24203647)

Supplementary Material

Figure S6: ^{1}H NMR, ^{13}C NMR, and HR-MS spectra of compound **1-15**.

Compound **1**

^{1}H NMR spectra of Compound **1**

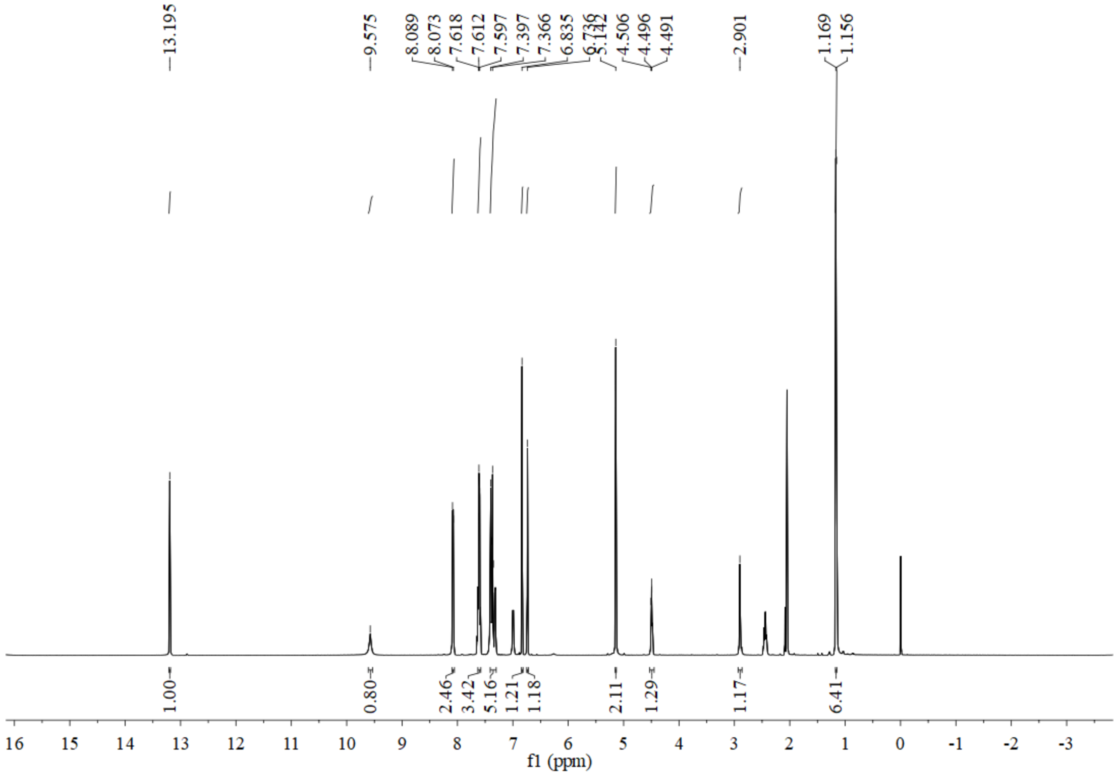

^{13}C NMR spectra of Compound **1**

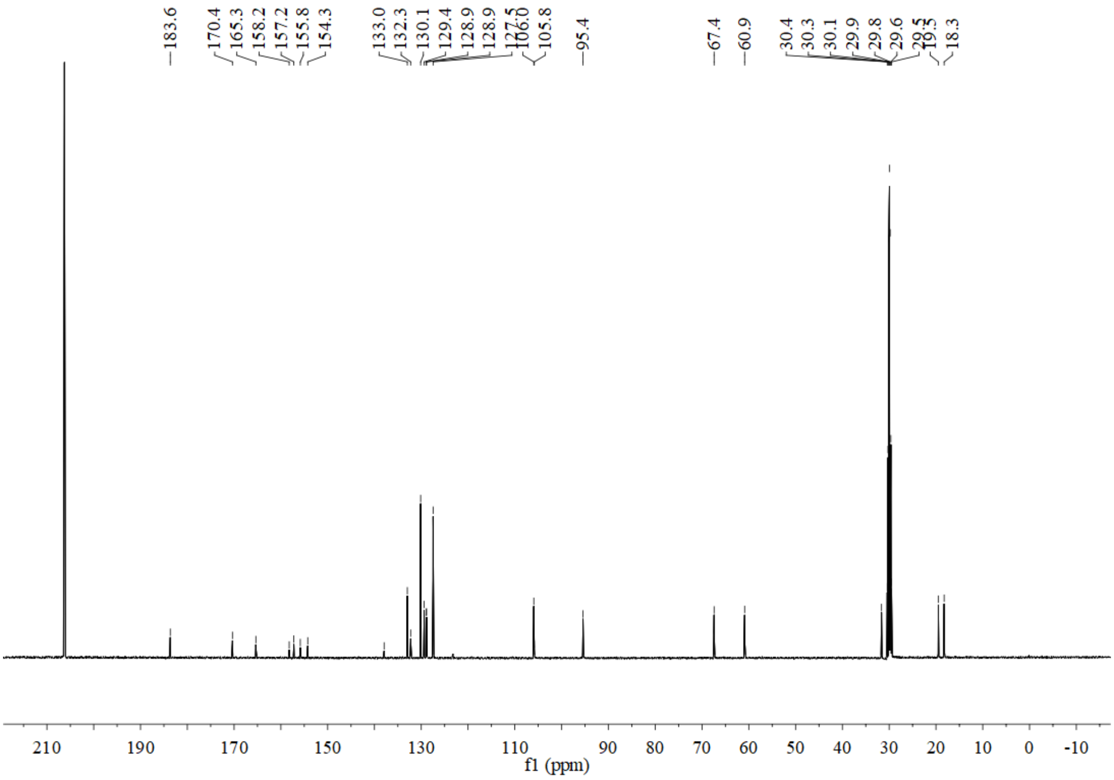

HR-MS of Compound **1**

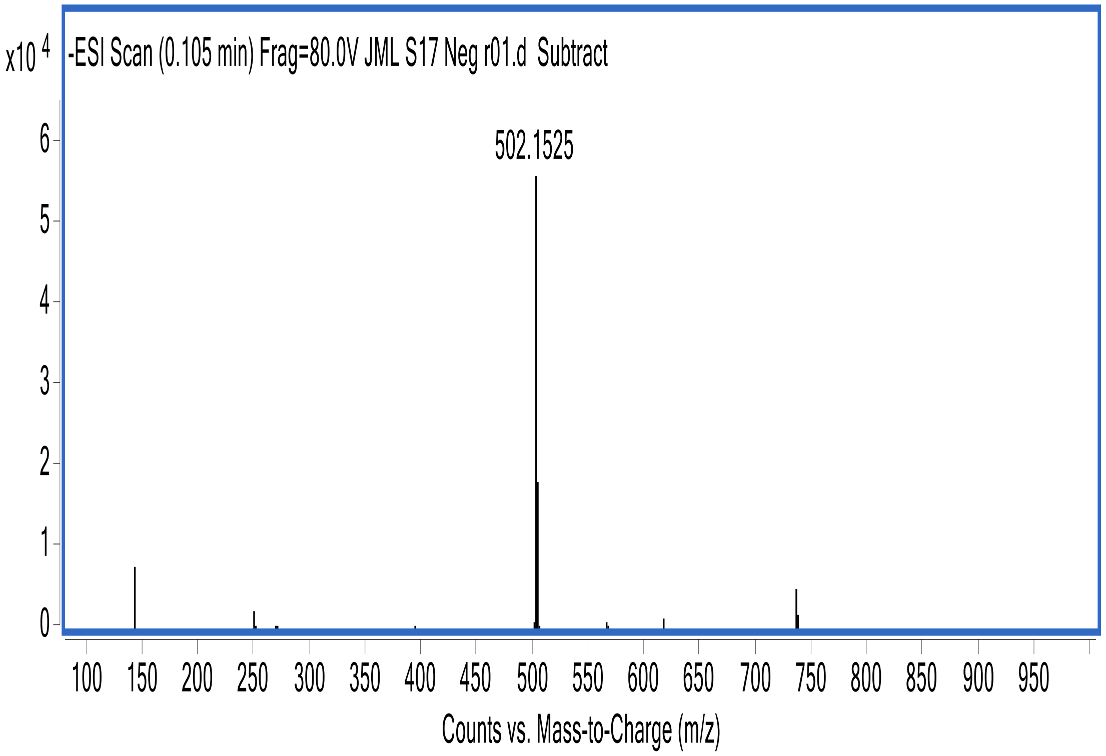

Compound **2**

^{1}H NMR spectra of Compound **2**

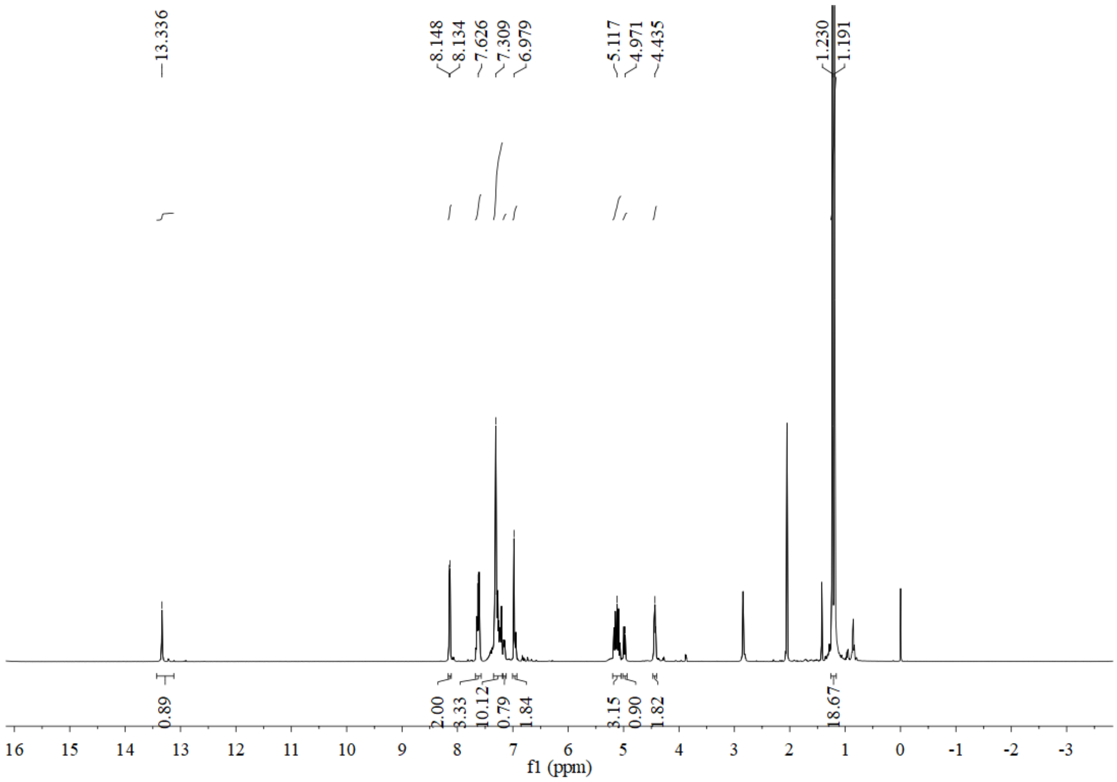

^{13}C NMR spectra of Compound **2**

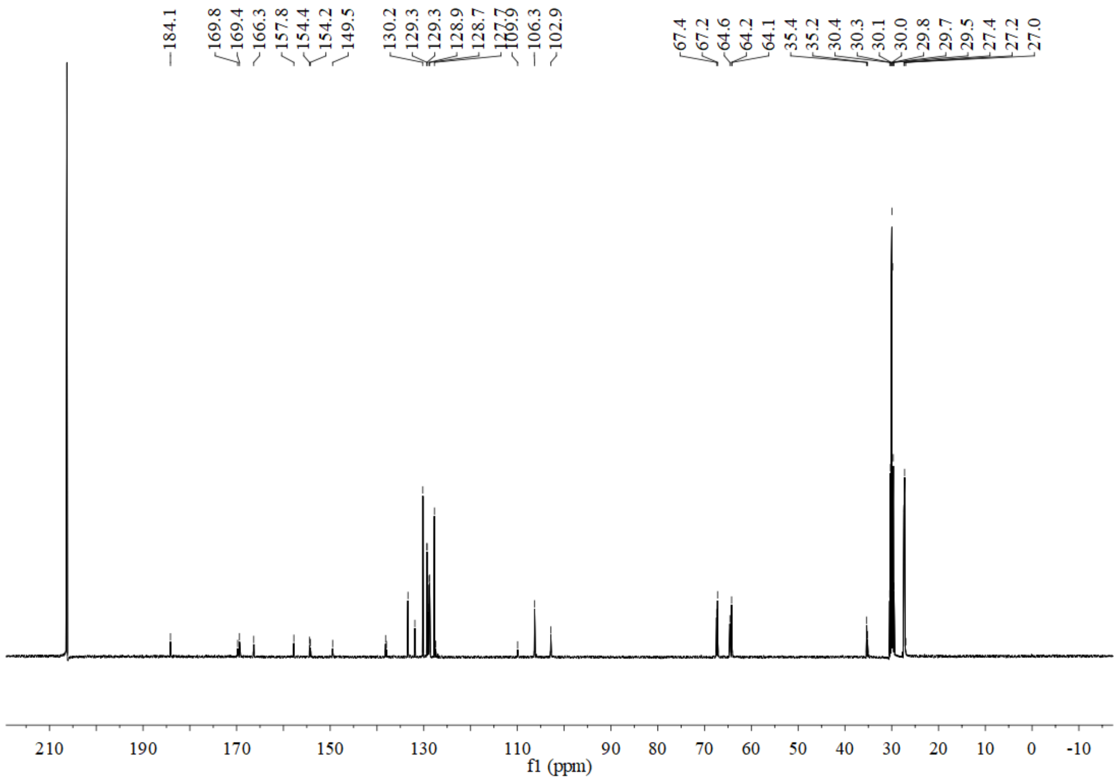

HR-MS of Compound **2**

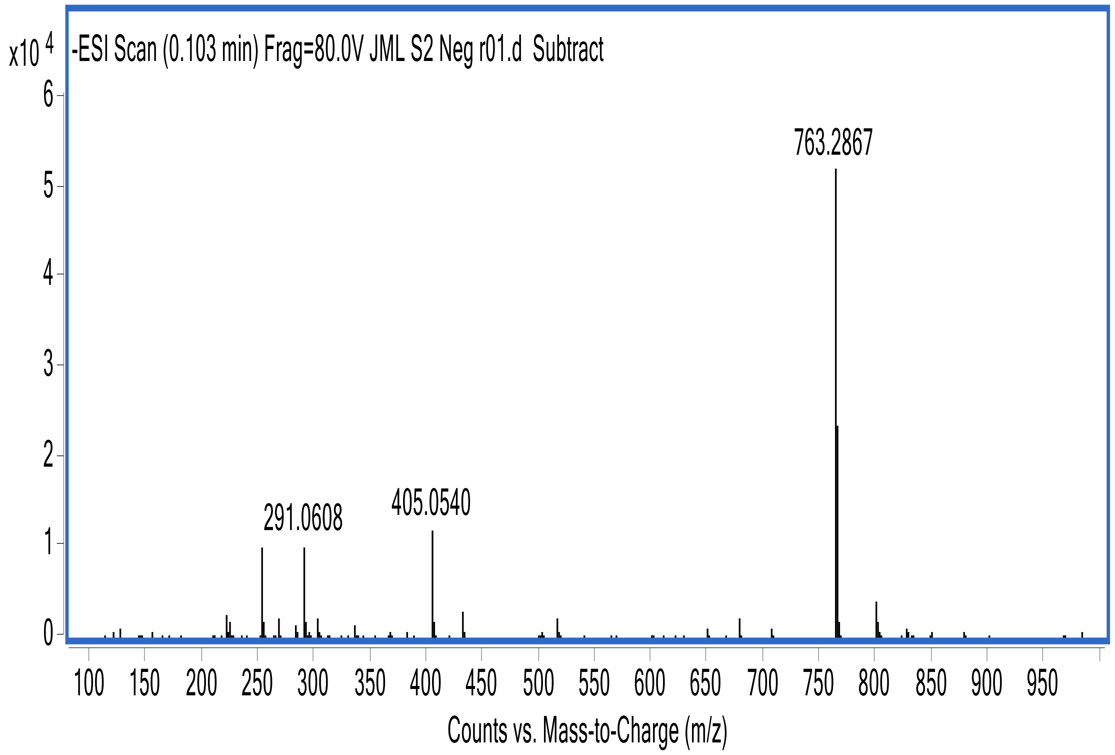

Compound **3**

^{1}H NMR spectra of Compound **3**

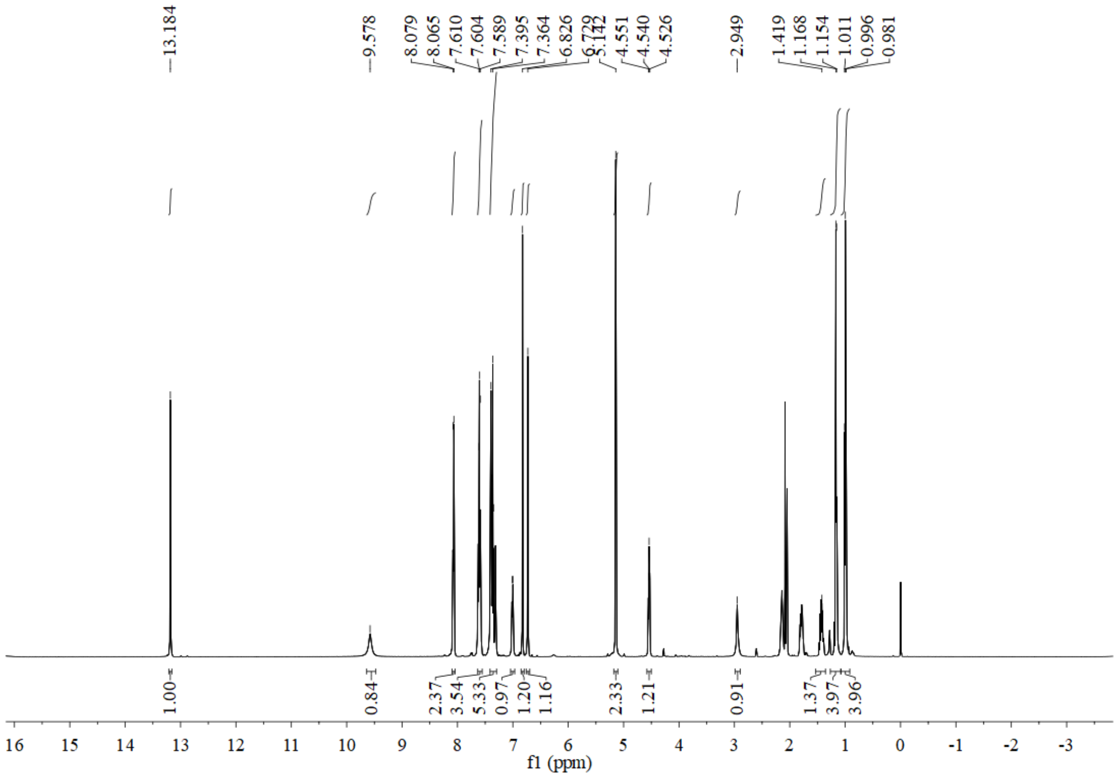

^{13}C NMR spectra of Compound **3**

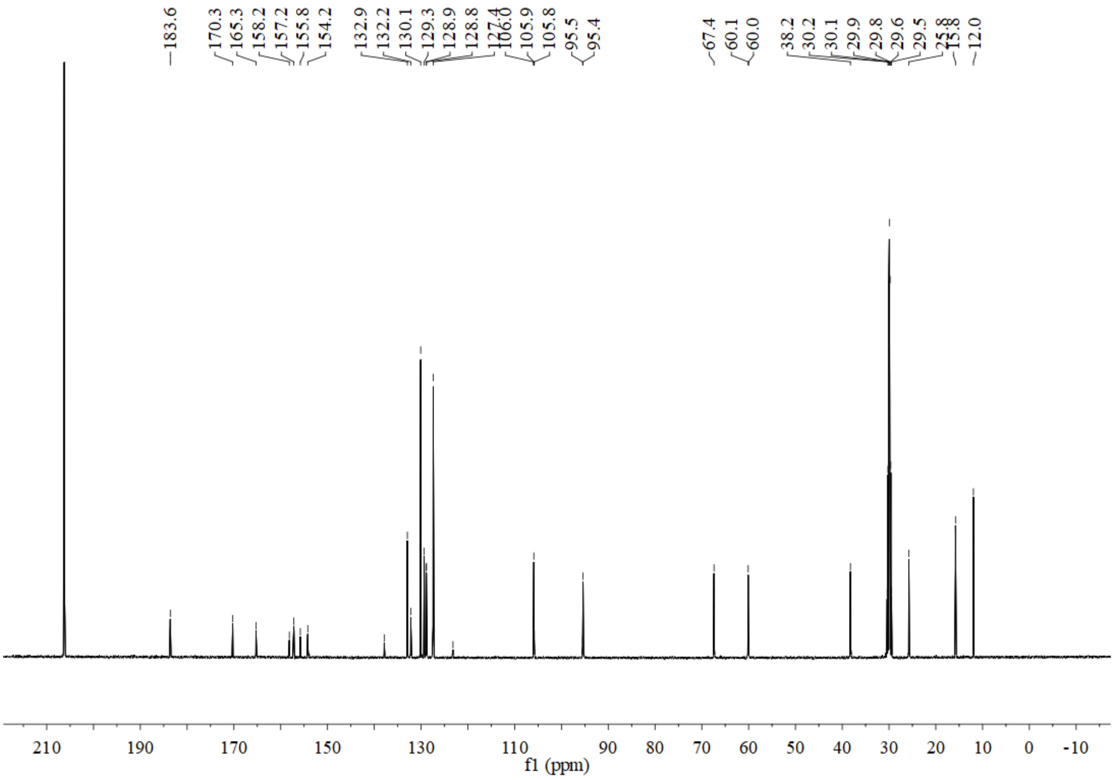

HR-MS of Compound **3**

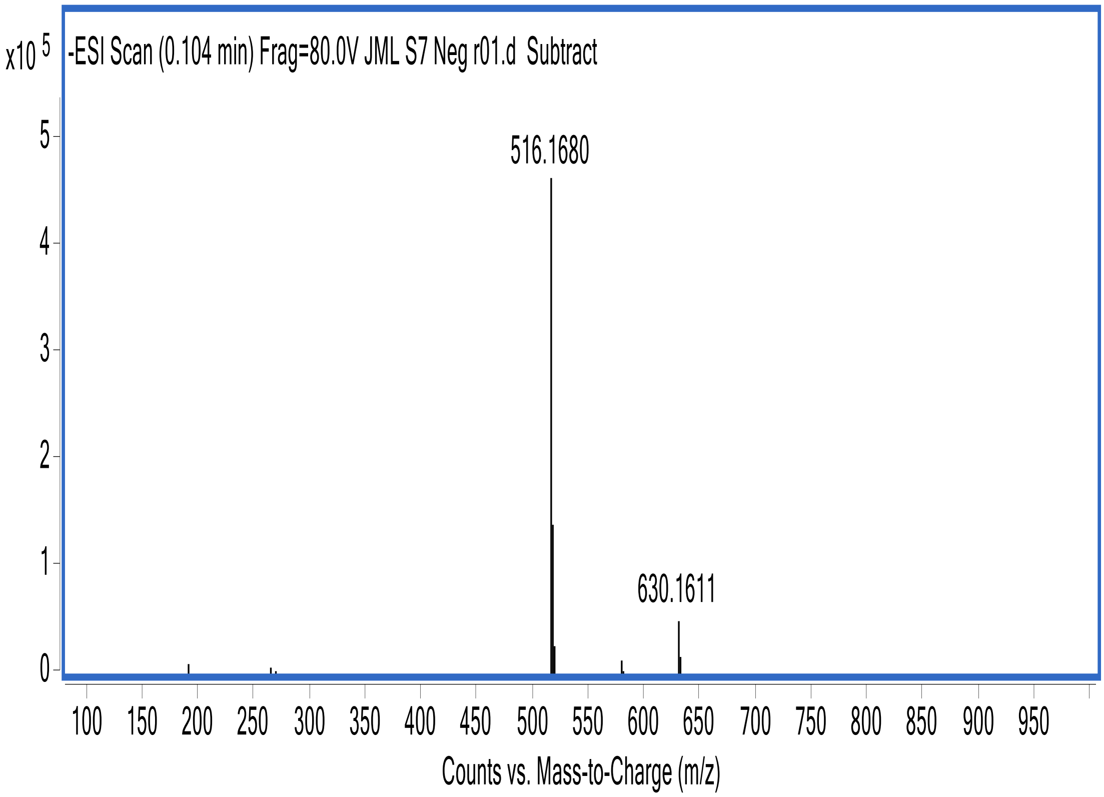

Compound **4**

^{1}H NMR spectra of Compound **4**

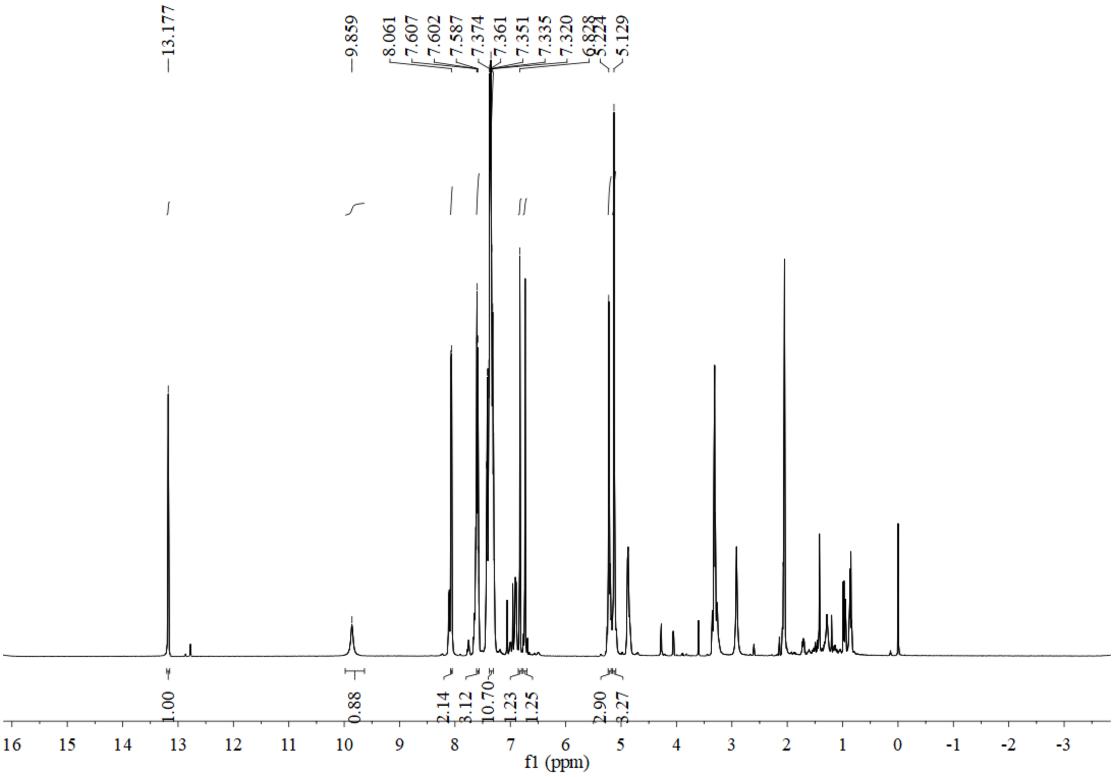

^{13}C NMR spectra of Compound **4**

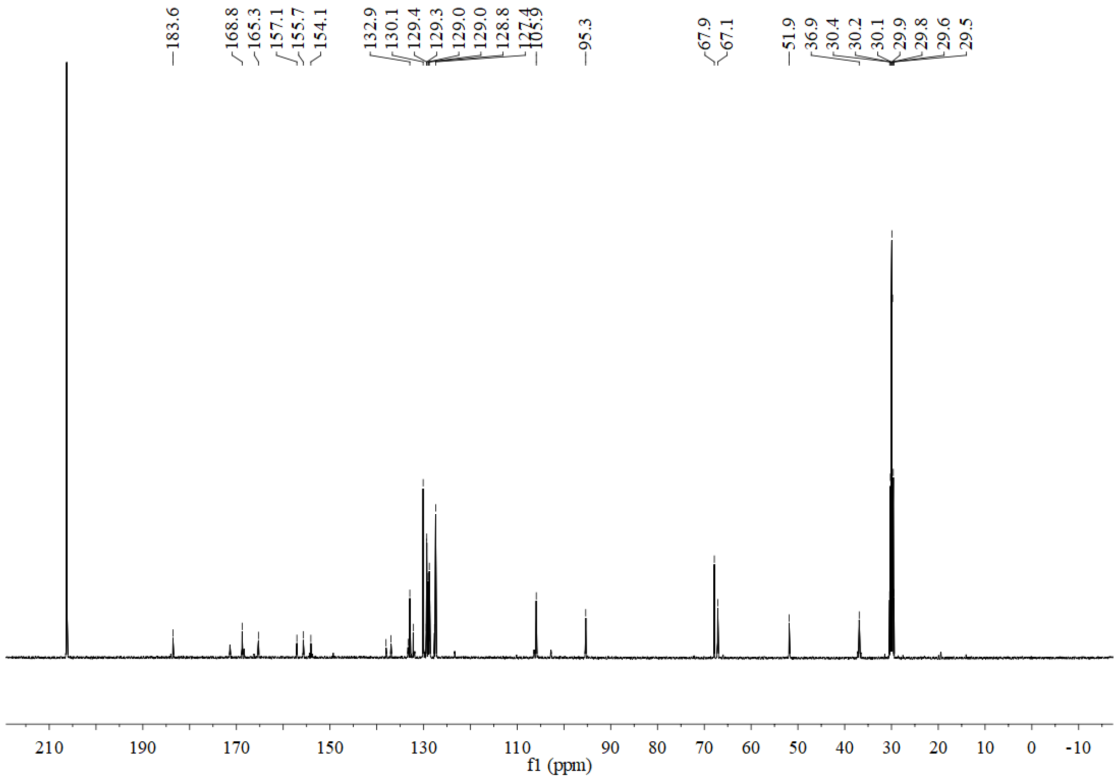

HR-MS of Compound **4**

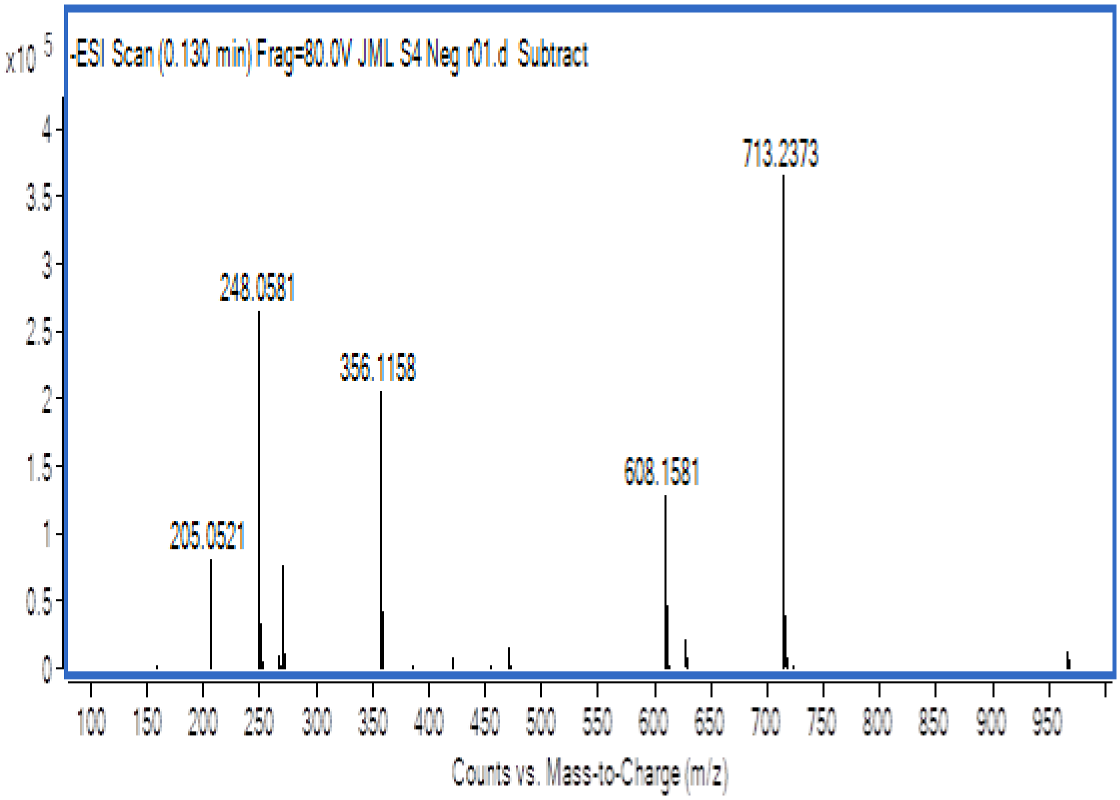

Compound **5**

^{1}H NMR spectra of Compound **5**

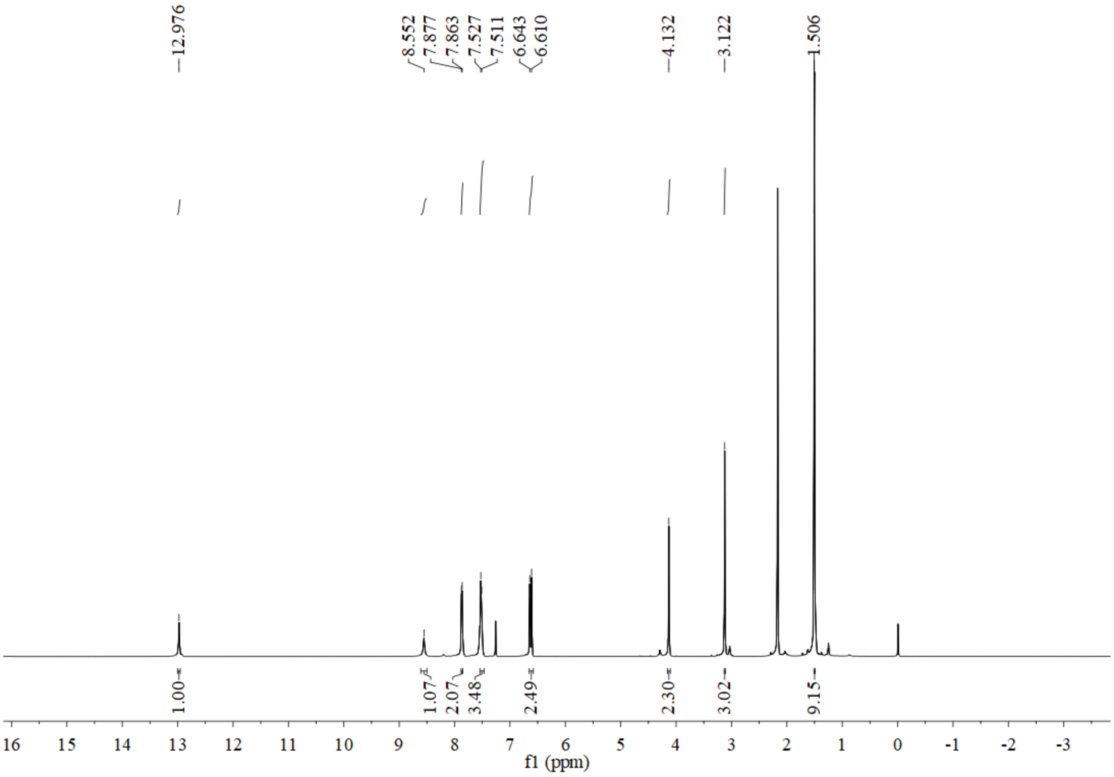

^{13}C NMR spectra of Compound **5**

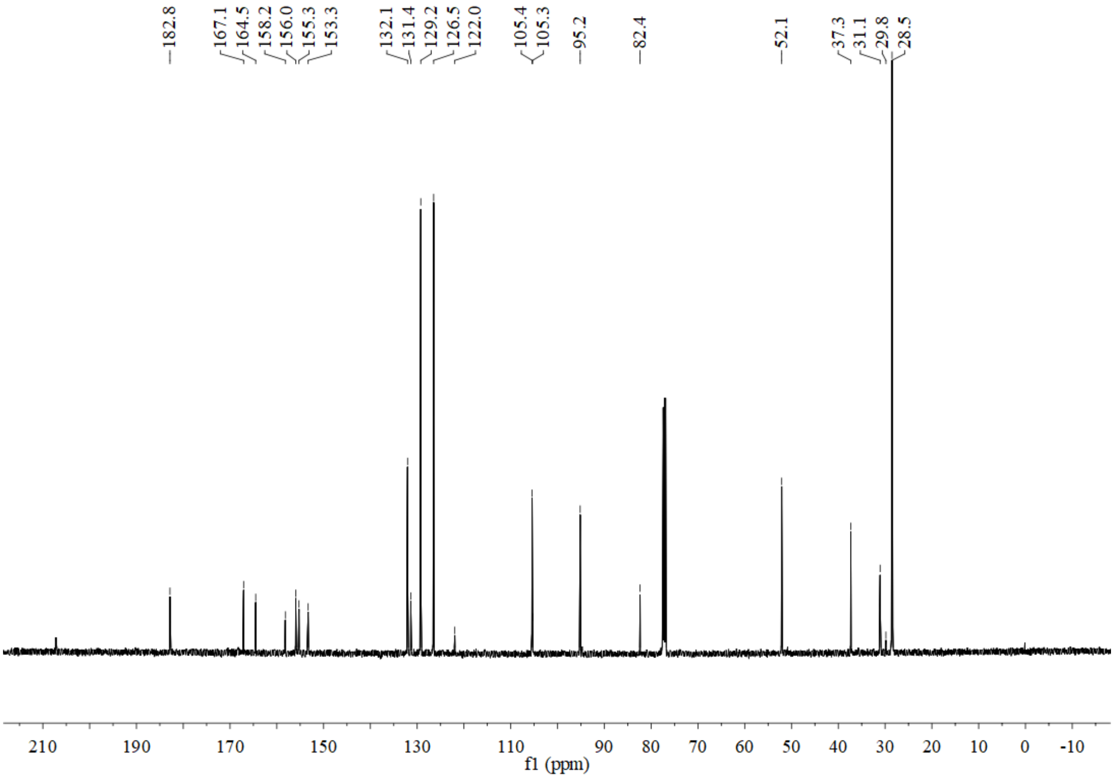

HR-MS of Compound **5**

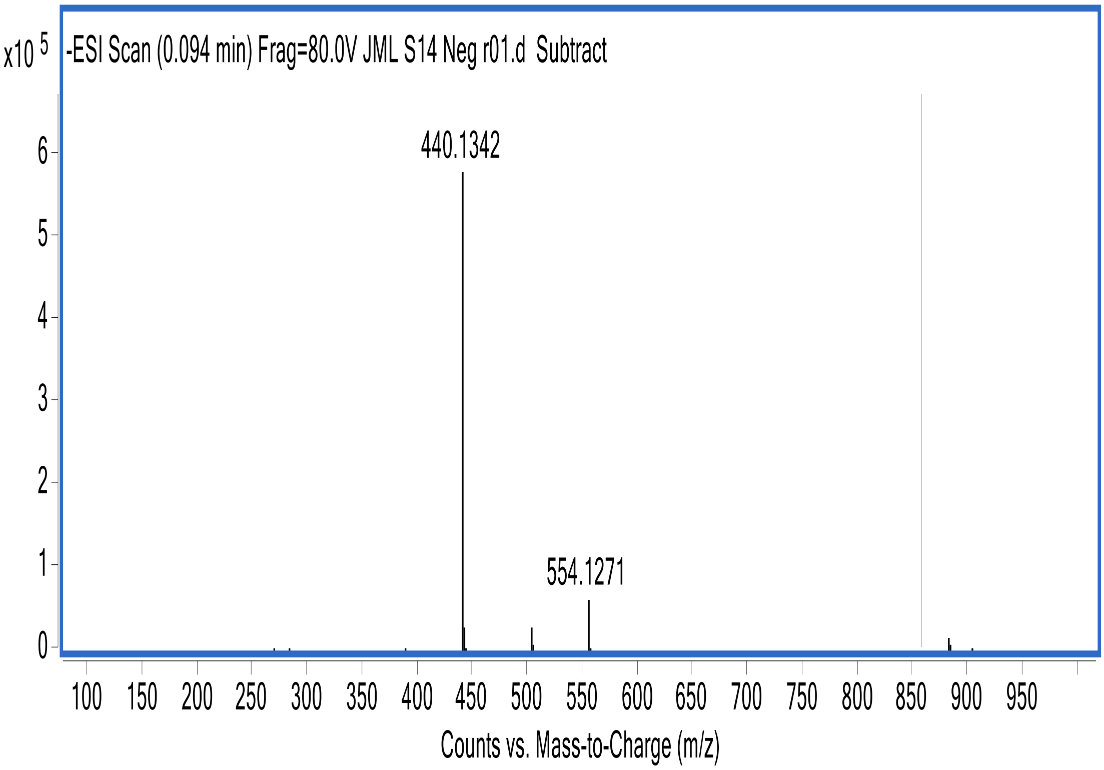

Compound **6**

^{1}H NMR spectra of Compound **6**

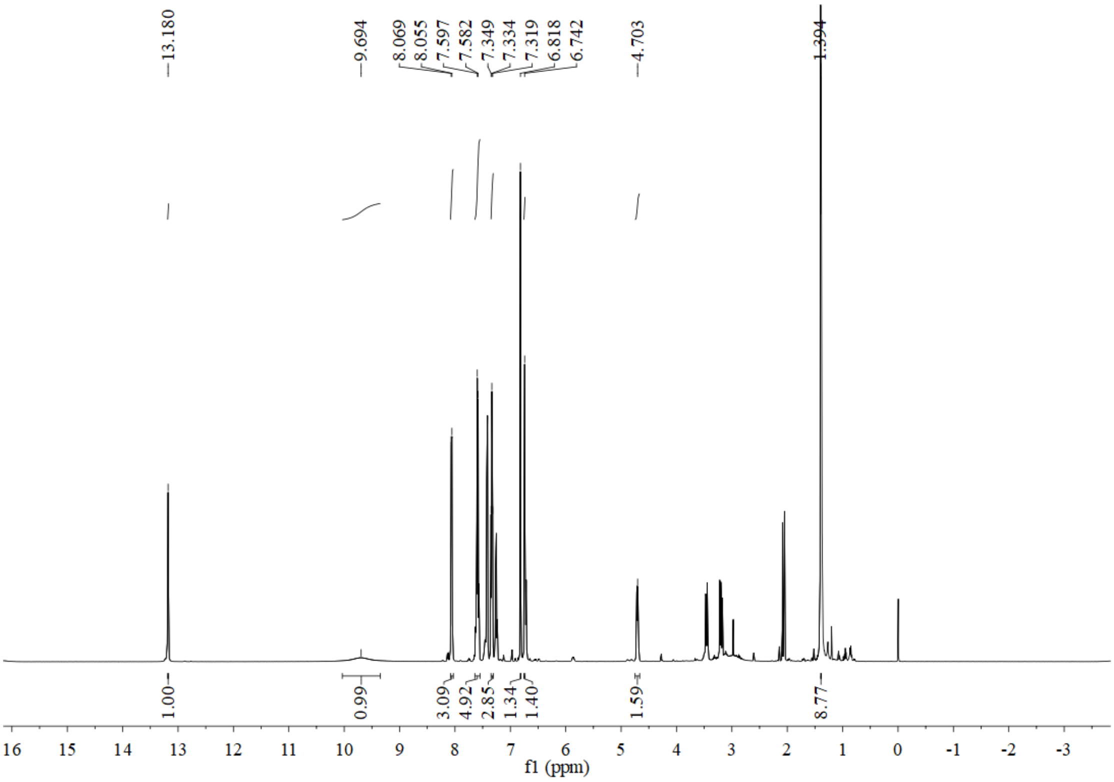

^{13}C NMR spectra of Compound **6**

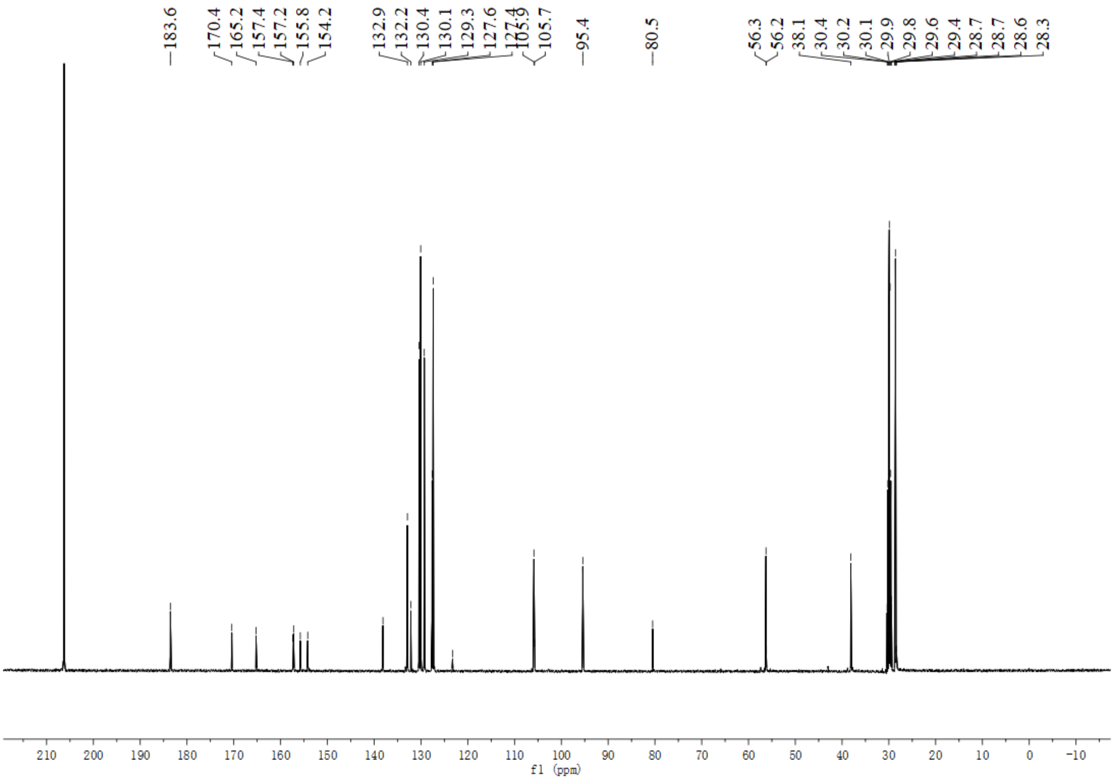

HR-MS of Compound **6**

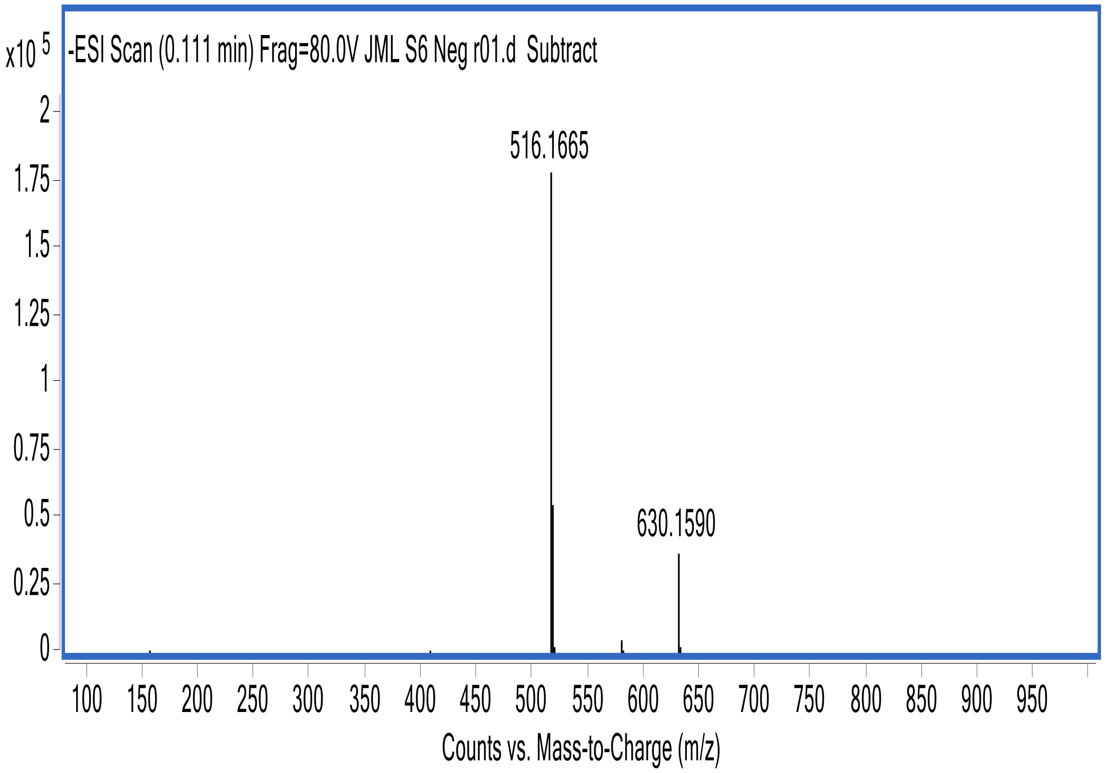

Compound **7**

^{1}H NMR spectra of Compound **7**

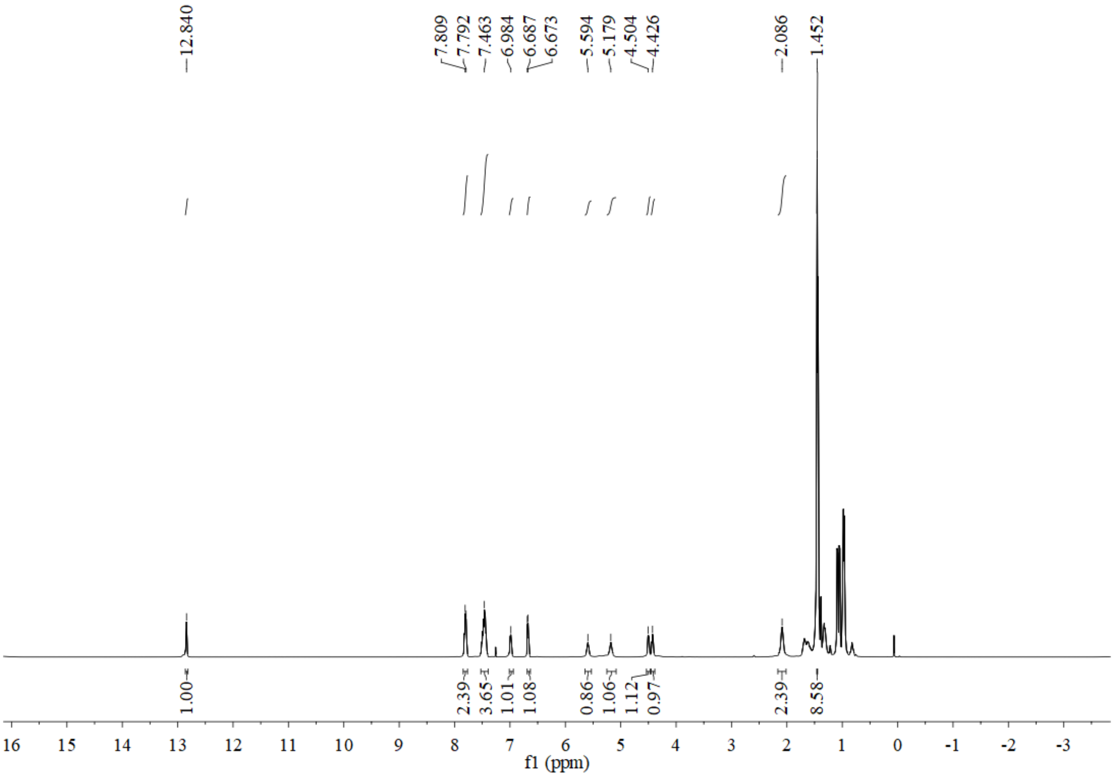

^{13}C NMR spectra of compound **7**

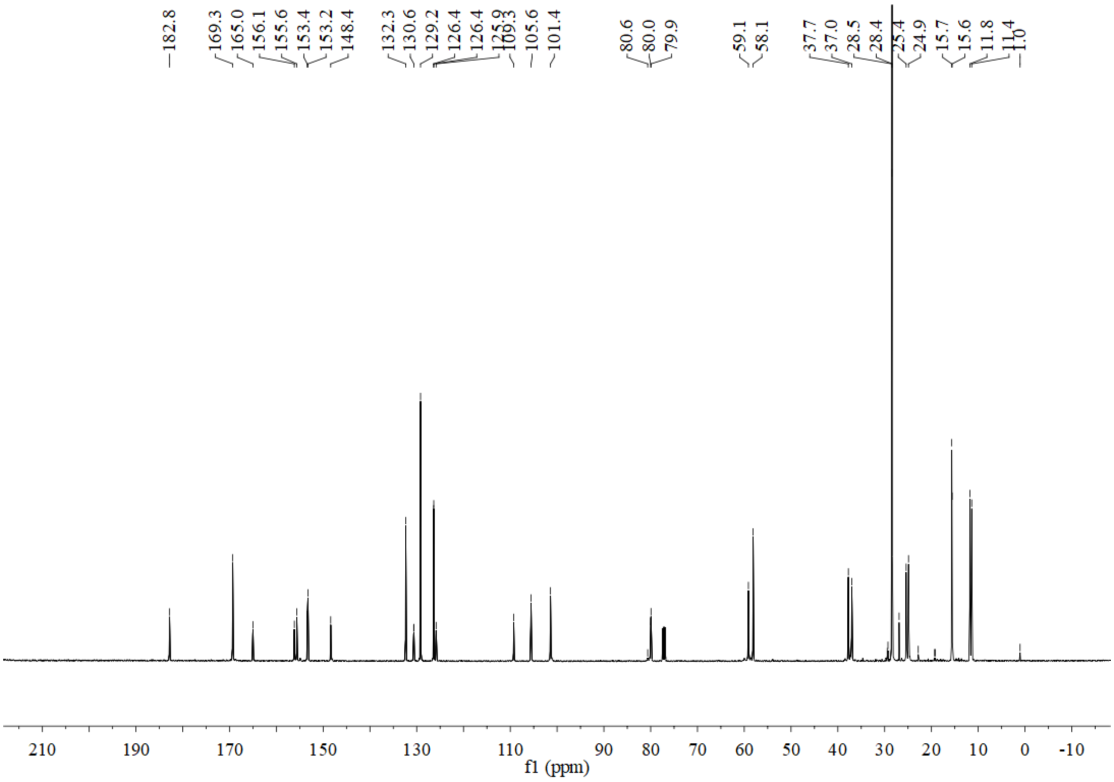

HR-MS of Compound **7**

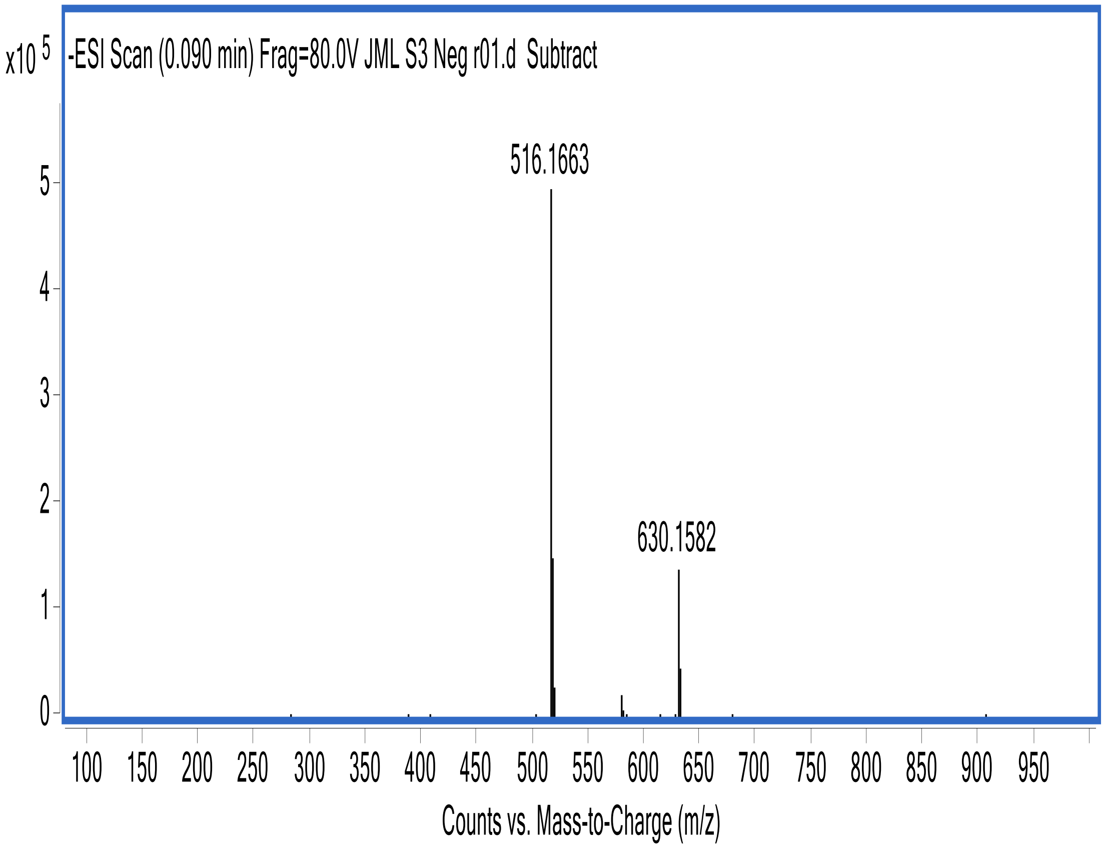

Compound **8**

^{1}H NMR spectra of compound **8**

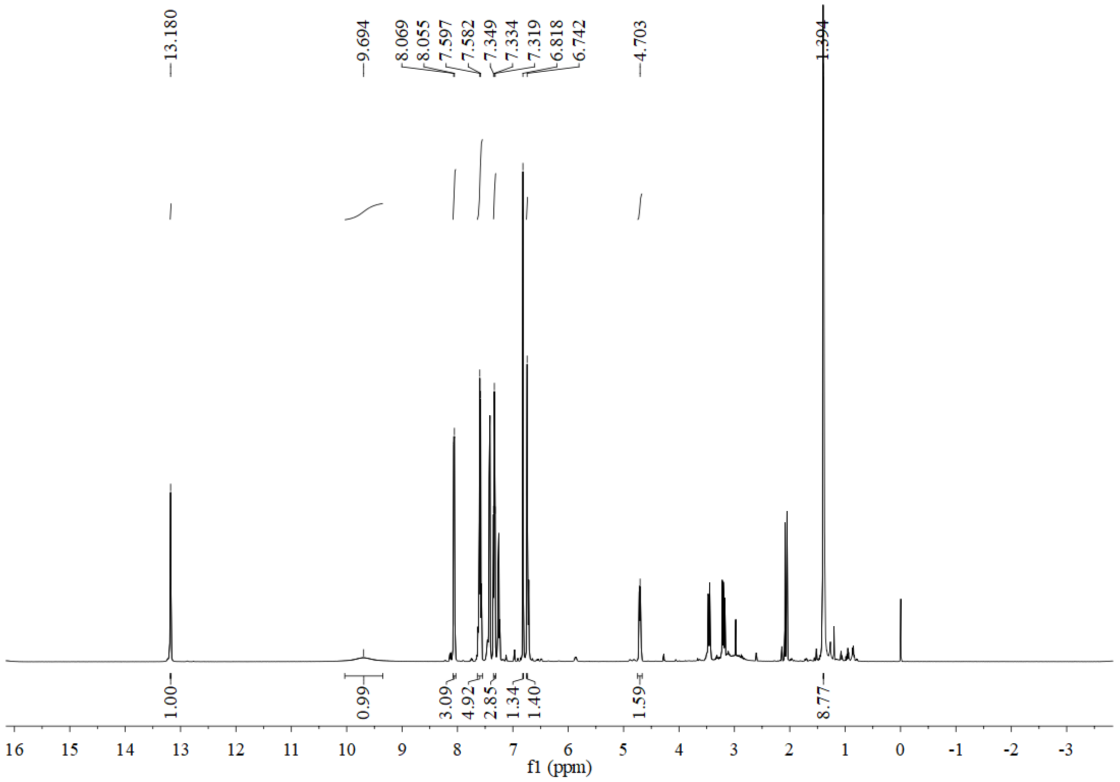

^{13}C NMR spectra of compound **8**

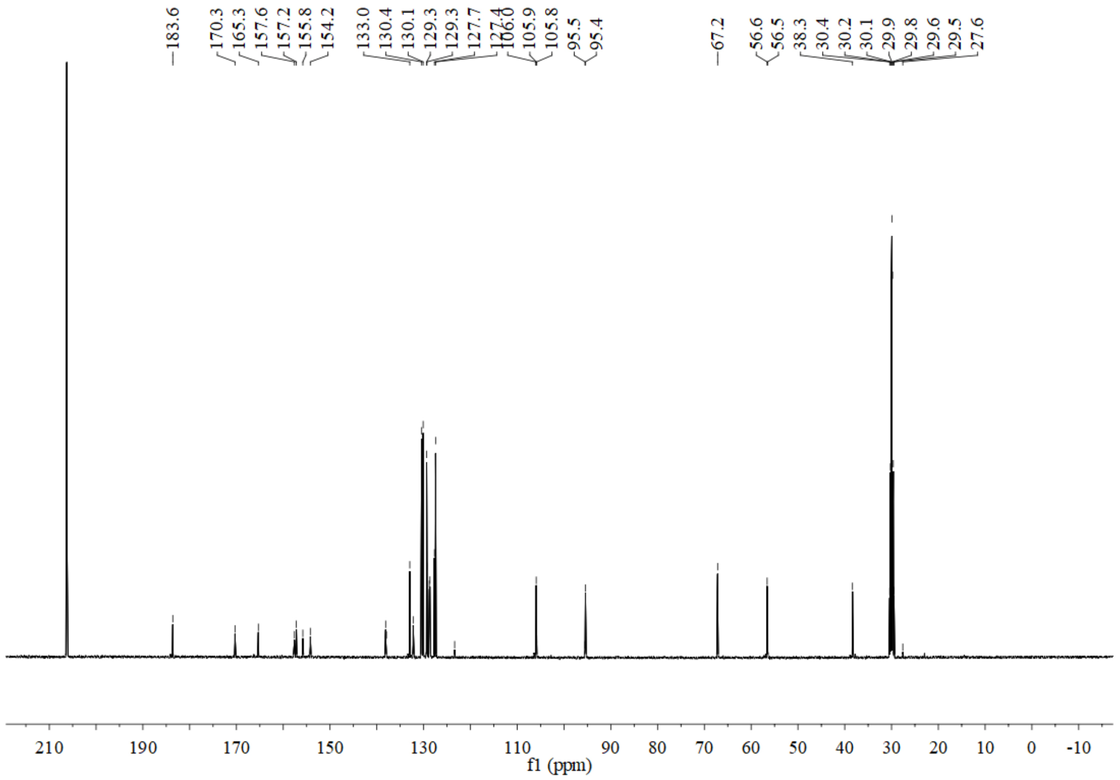

HR-MS of Compound **8**

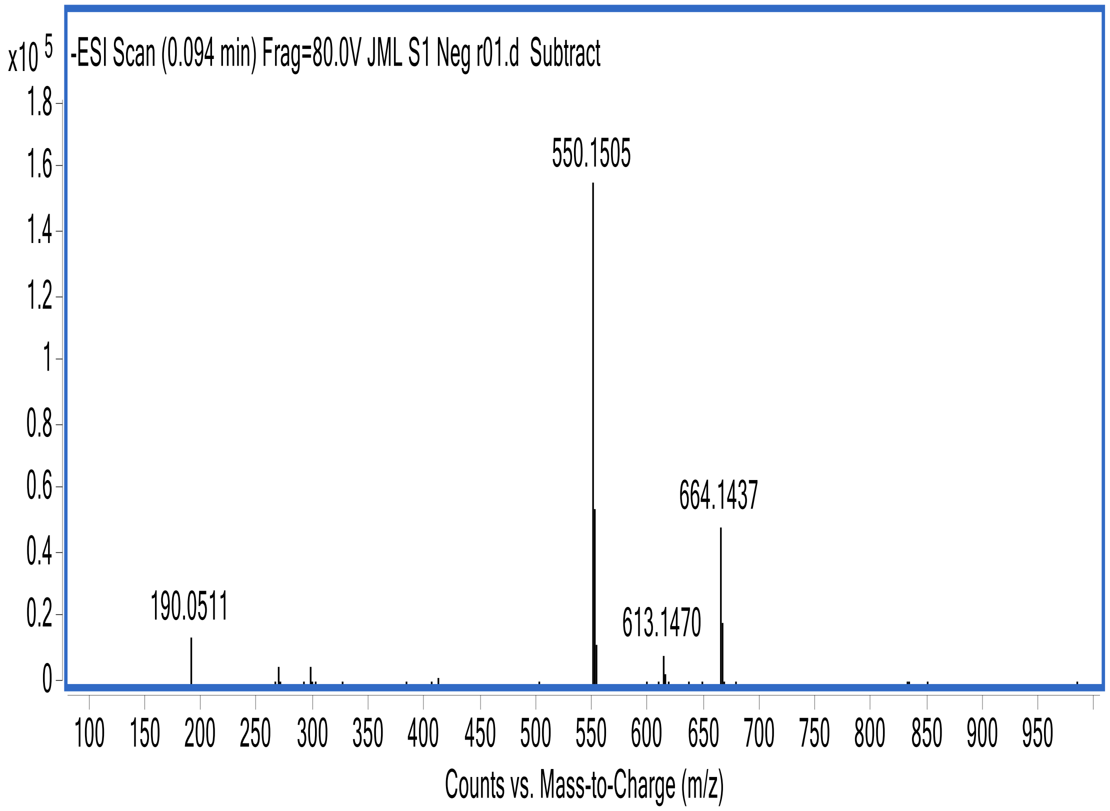

Compound **9**

^{1}H NMR spectra of compound **9**

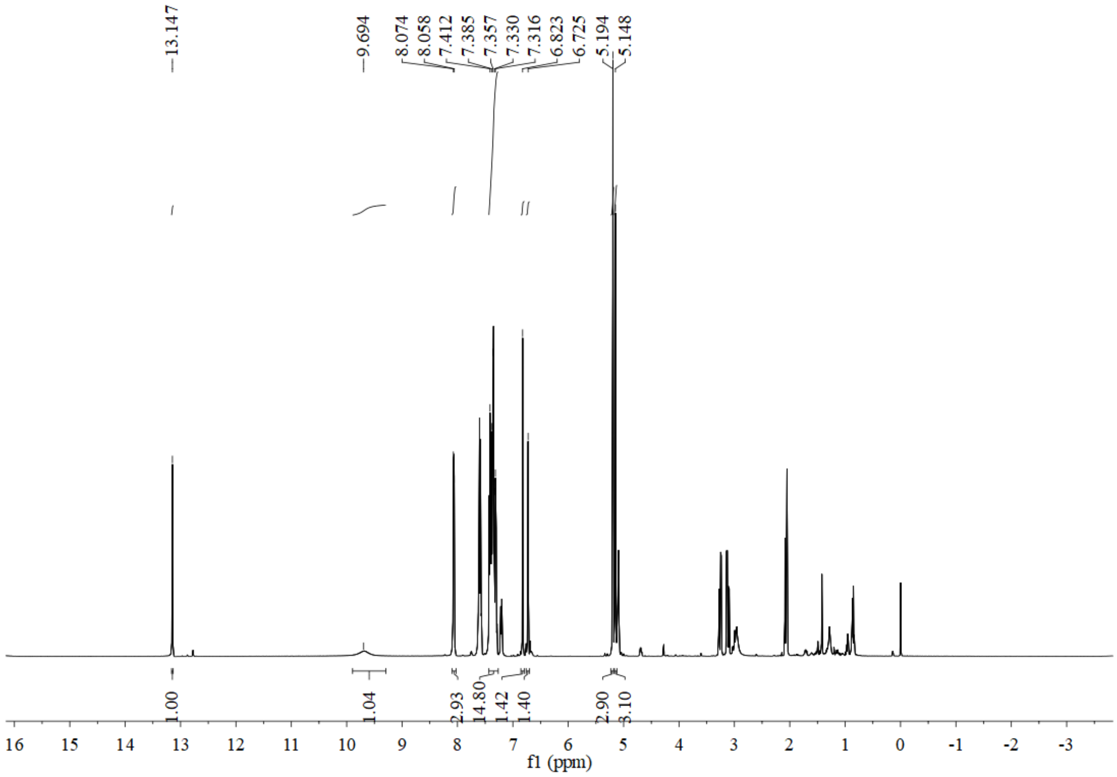

^{13}C NMR spectra of compound **9**

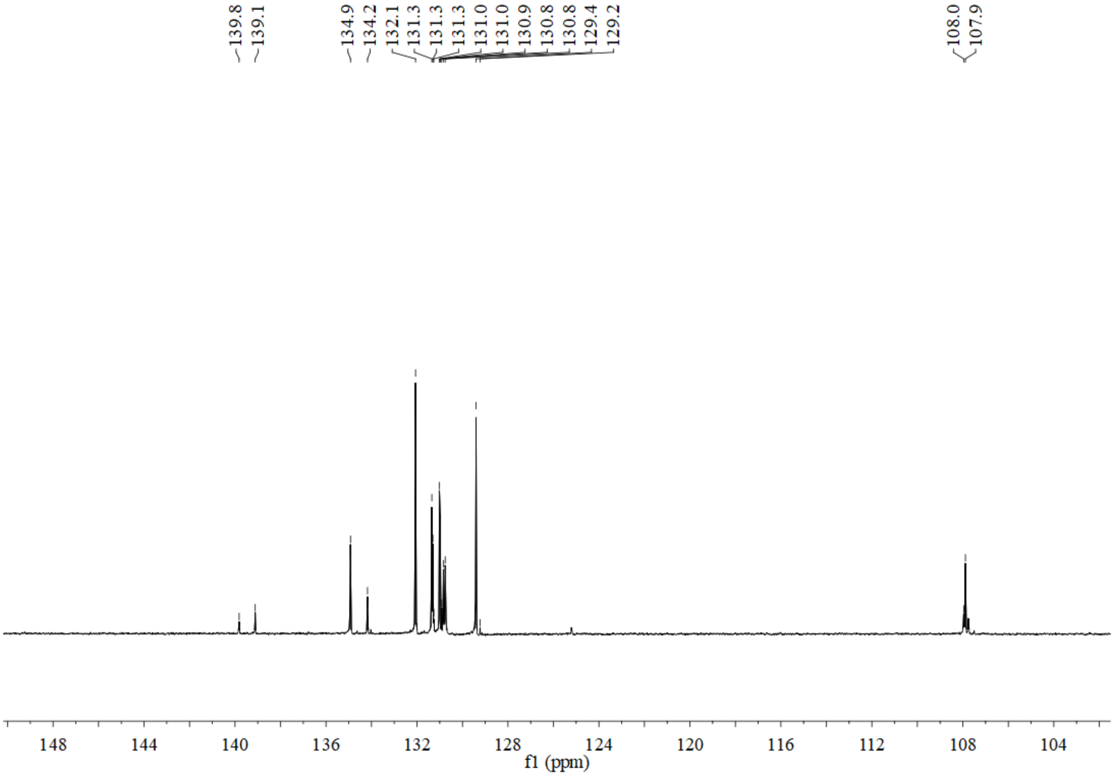

HR-MS of Compound **9**

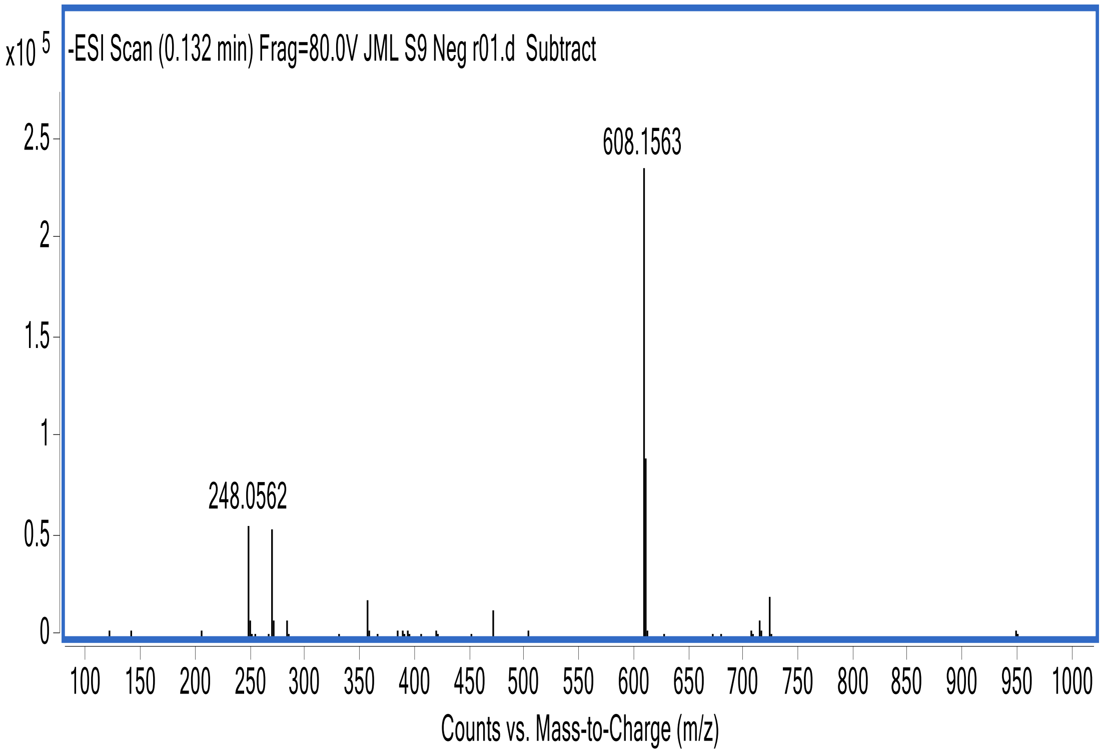

Compound **10**

^{1}H NMR spectra of compound **10**

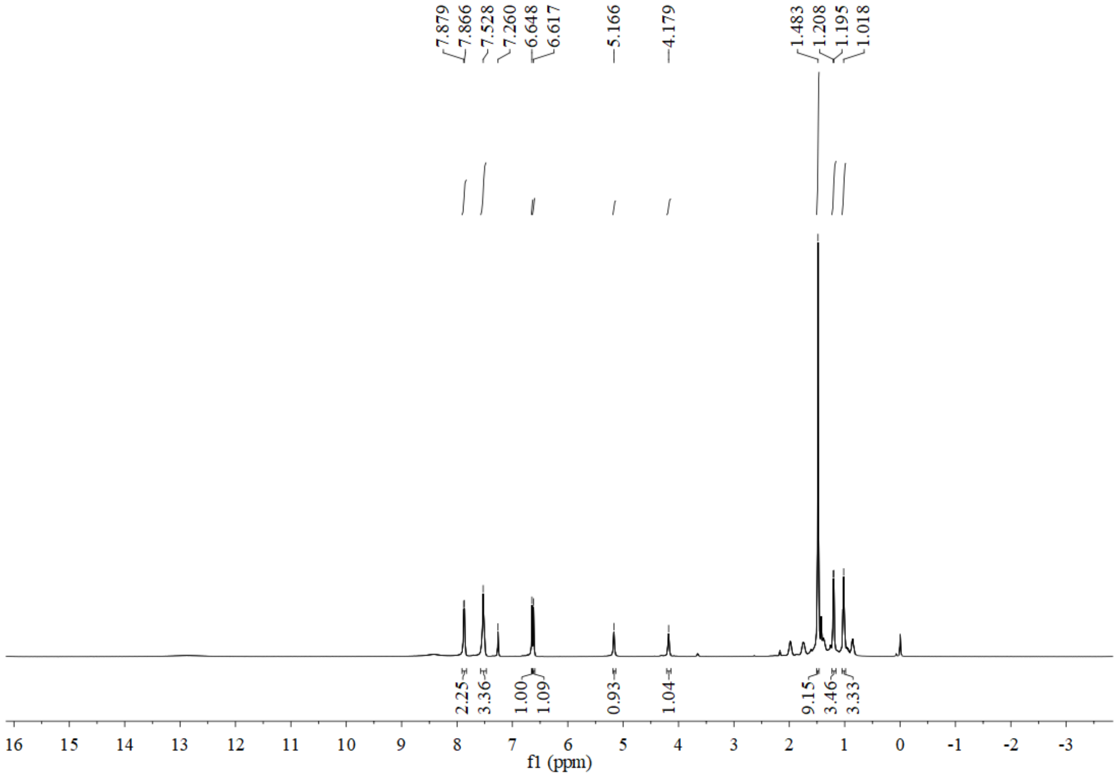

^{13}C NMR spectra of compound **10**

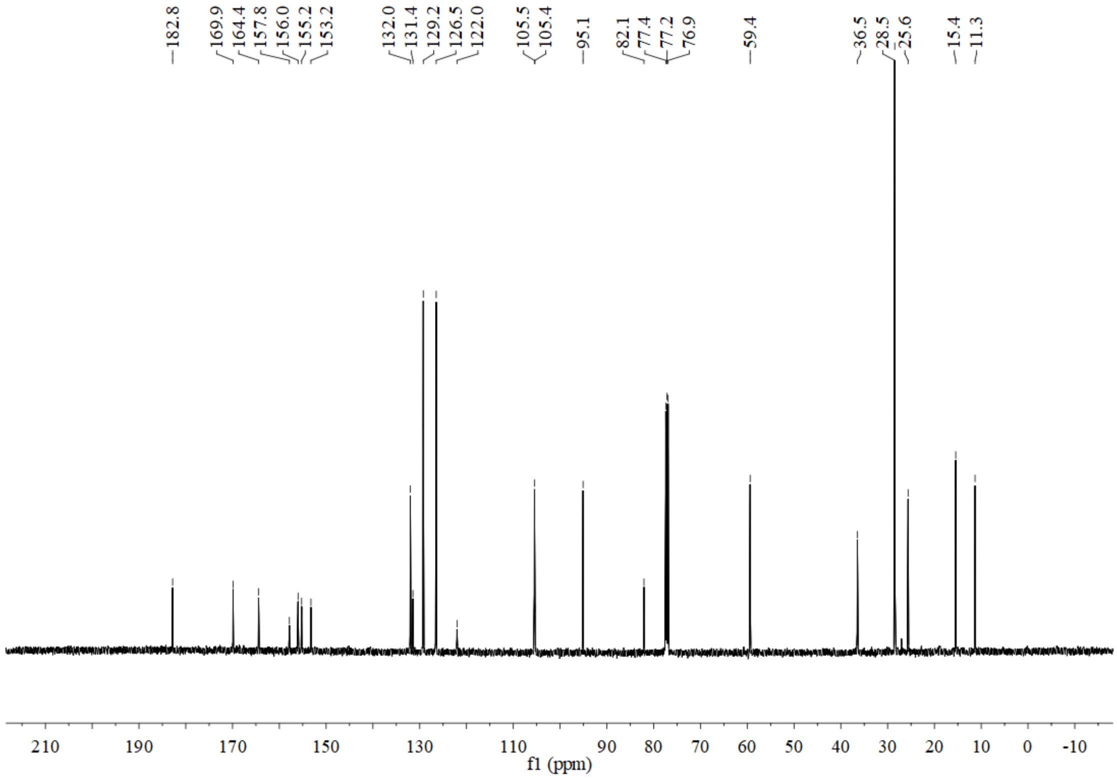

HR-MS of Compound **10**

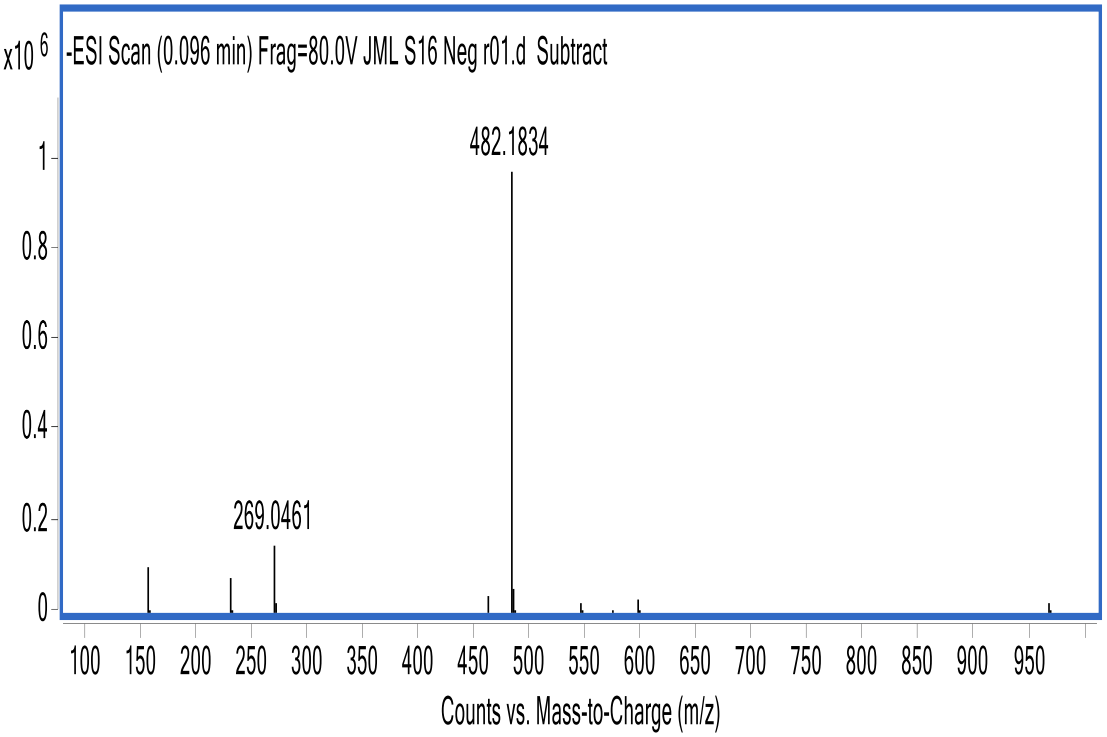

Compound **11**

^{1}H NMR spectra of compound **11**

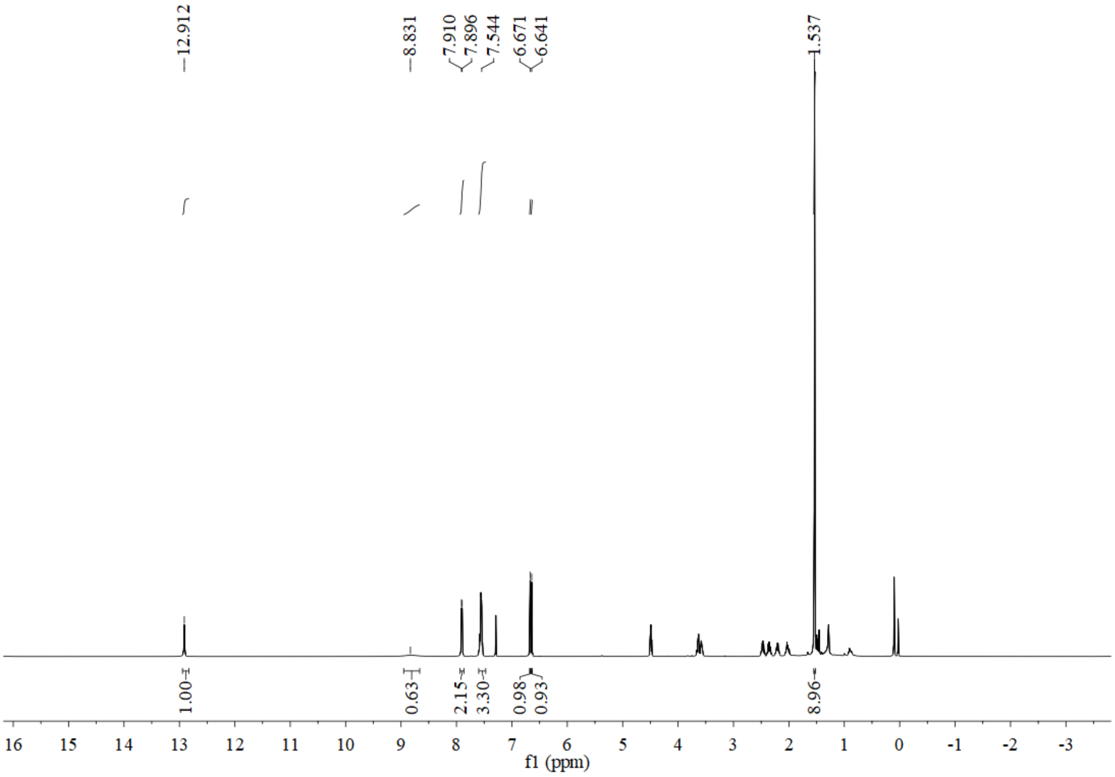

^{13}C NMR spectra of compound **11**

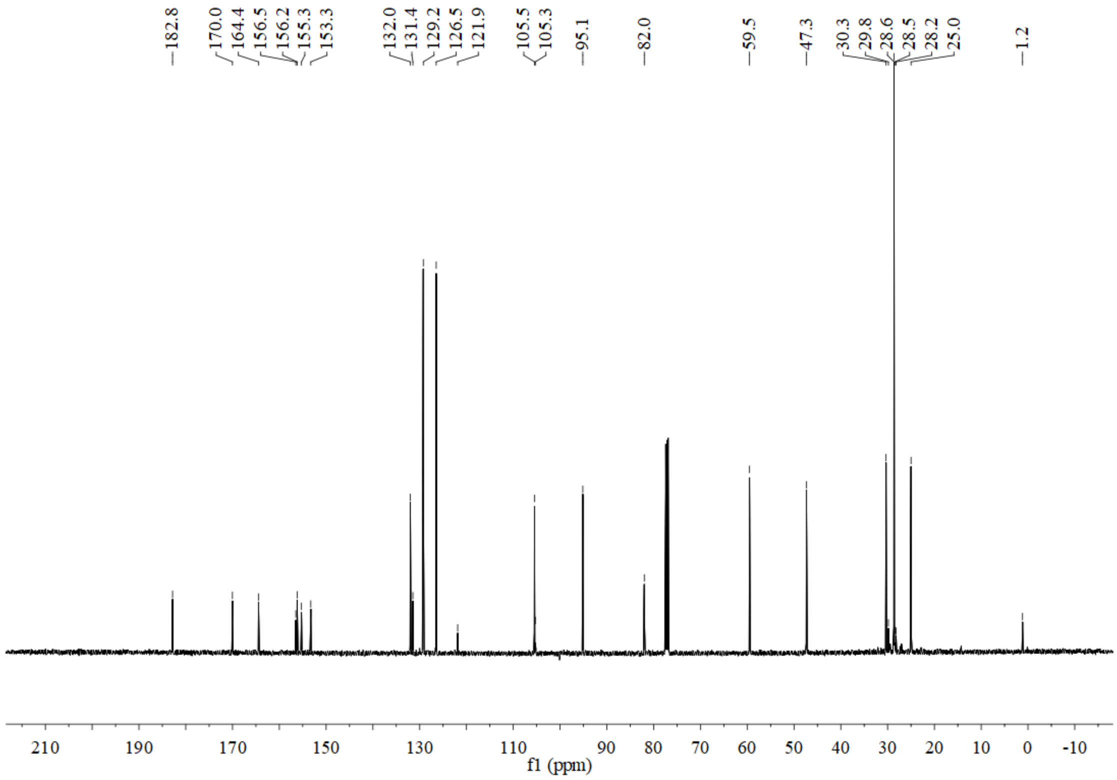

HR-MS of Compound **11**

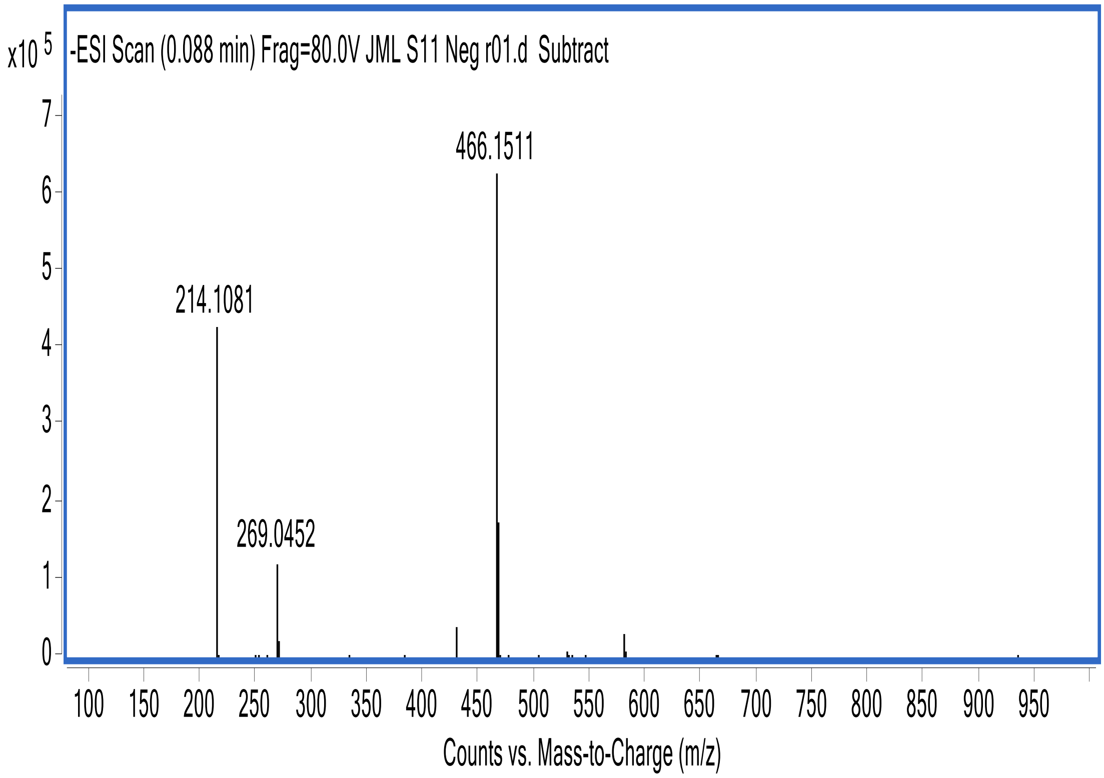

Compound **12**

^{1}H NMR spectra of compound **12**

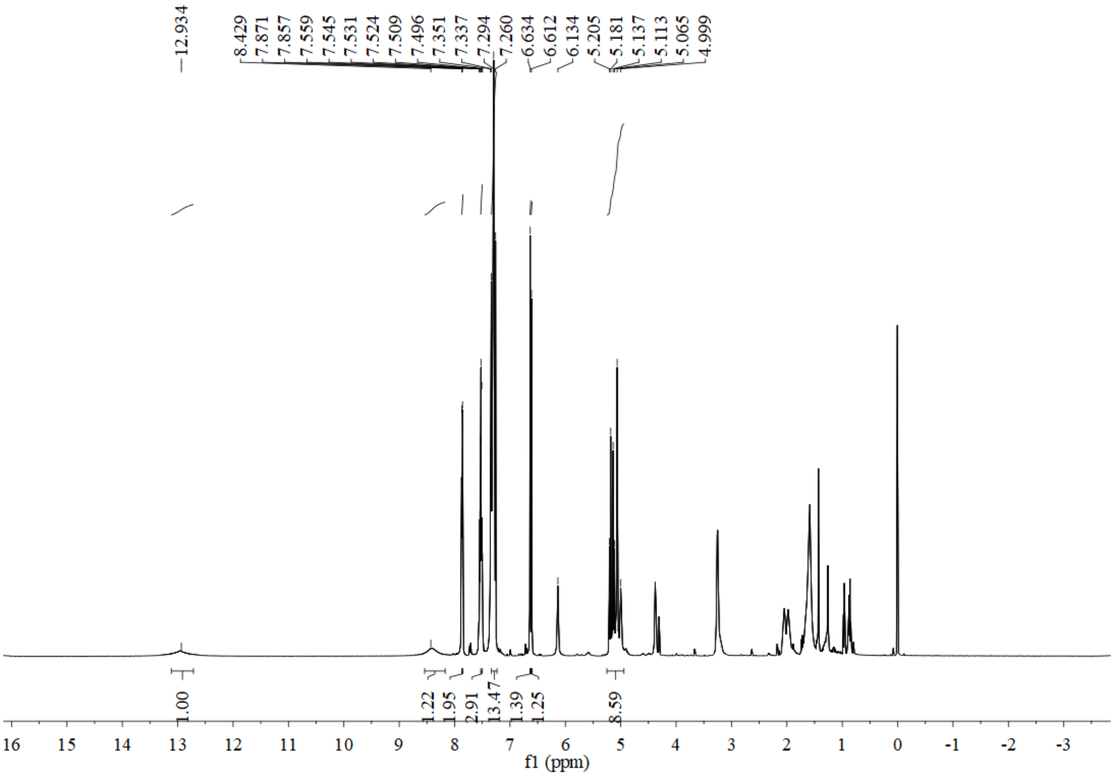

^{13}C NMR spectra of compound **12**

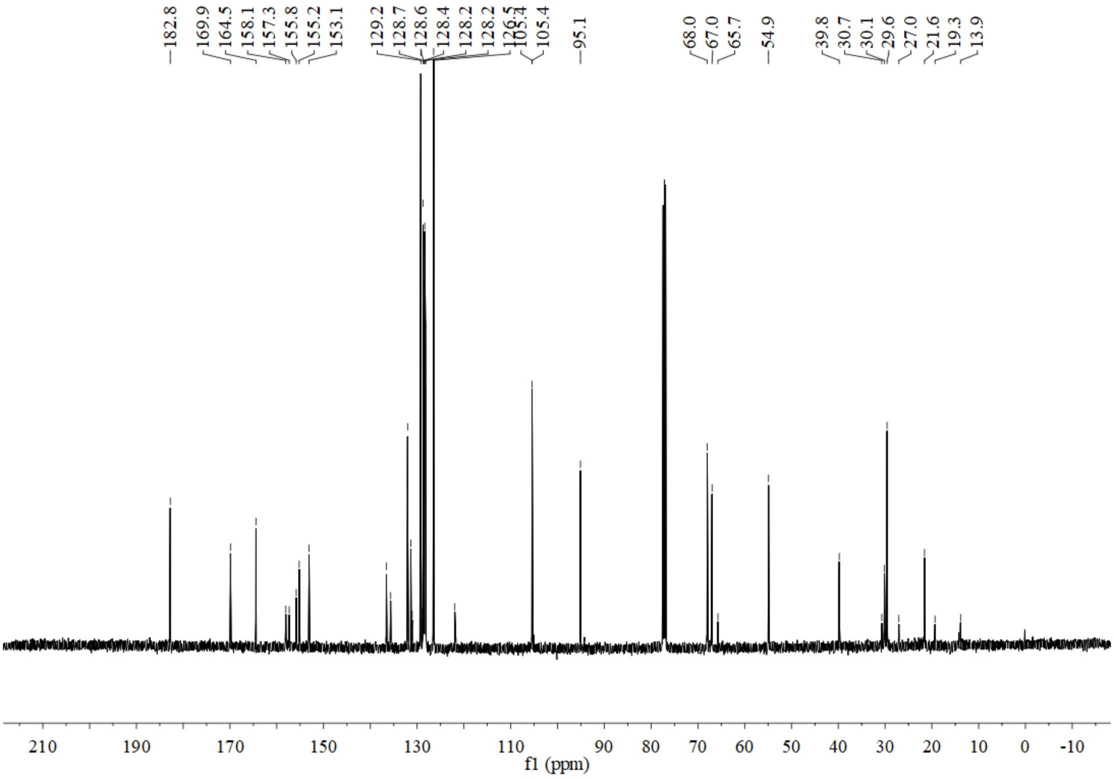

HR-MS of Compound **12**

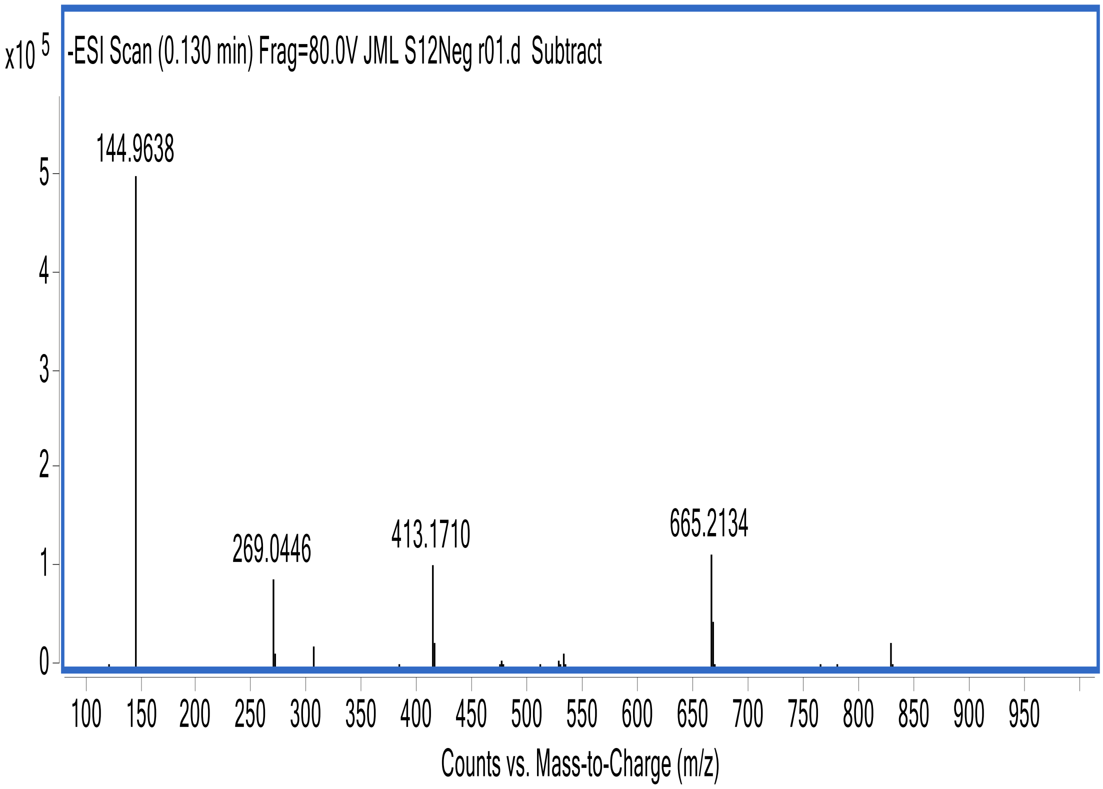

Compound **13**

^{1}H NMR spectra of compound **13**

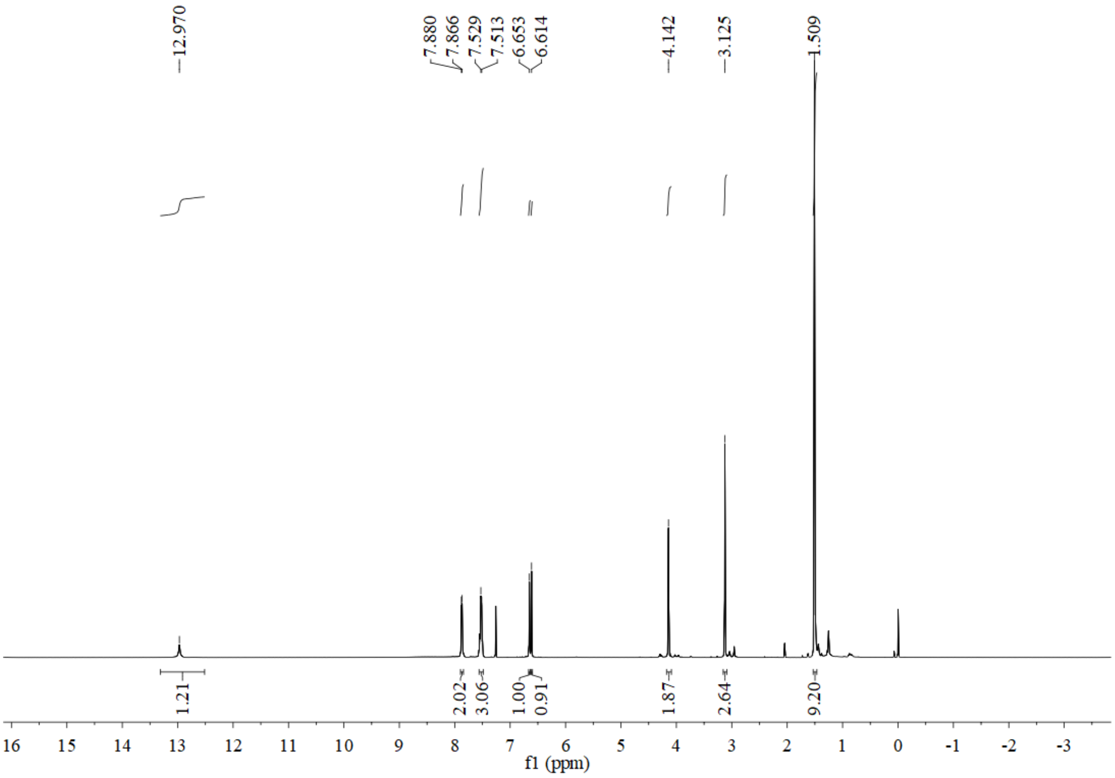

^{13}C NMR spectra of compound **13**

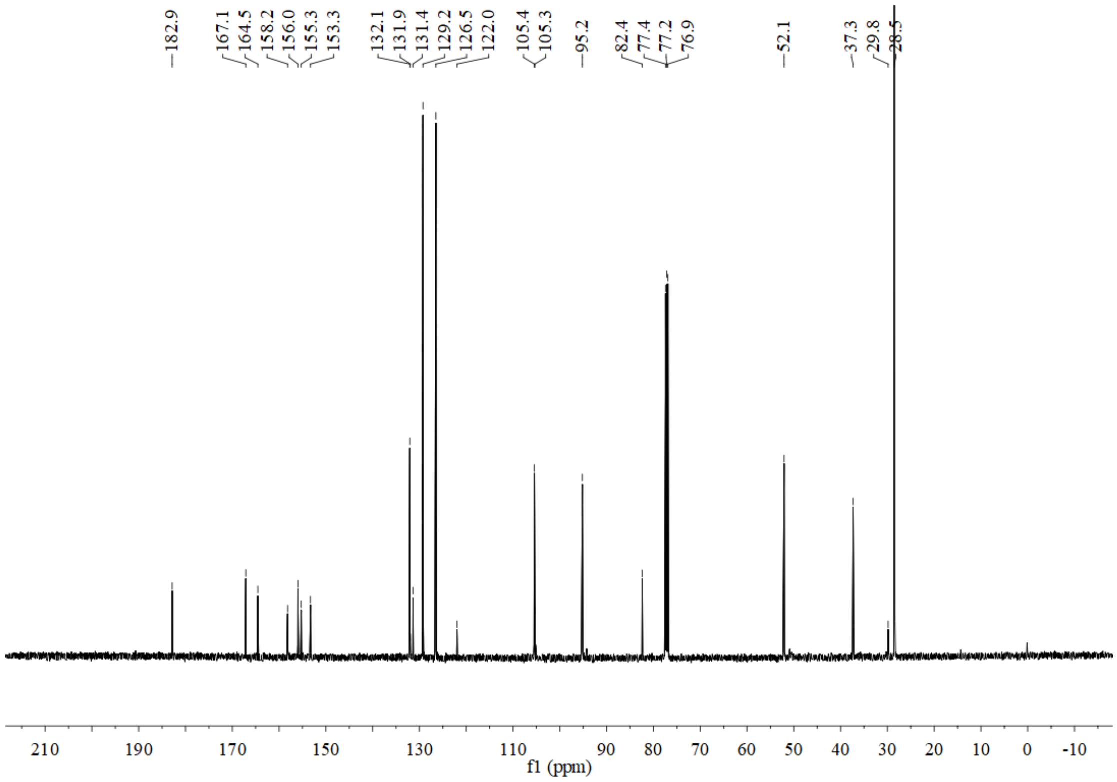

HR-MS of Compound **13**

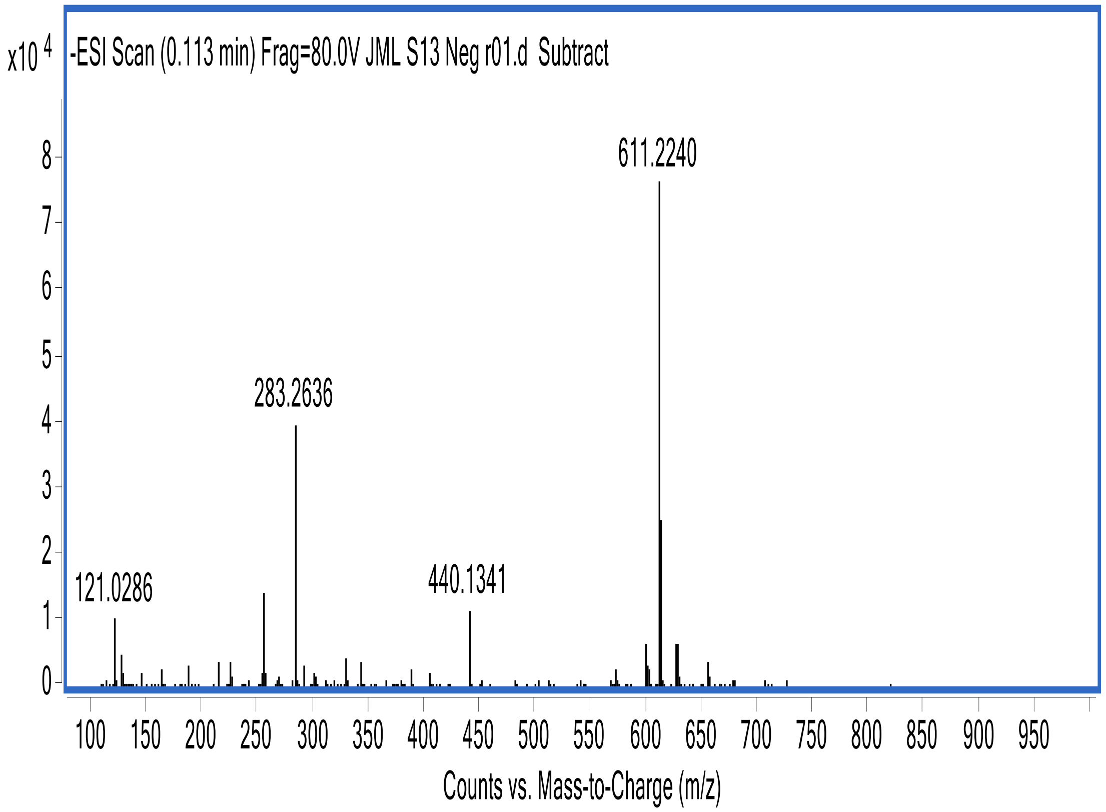

Compound **14**

^{1}H NMR spectra of compound **14**

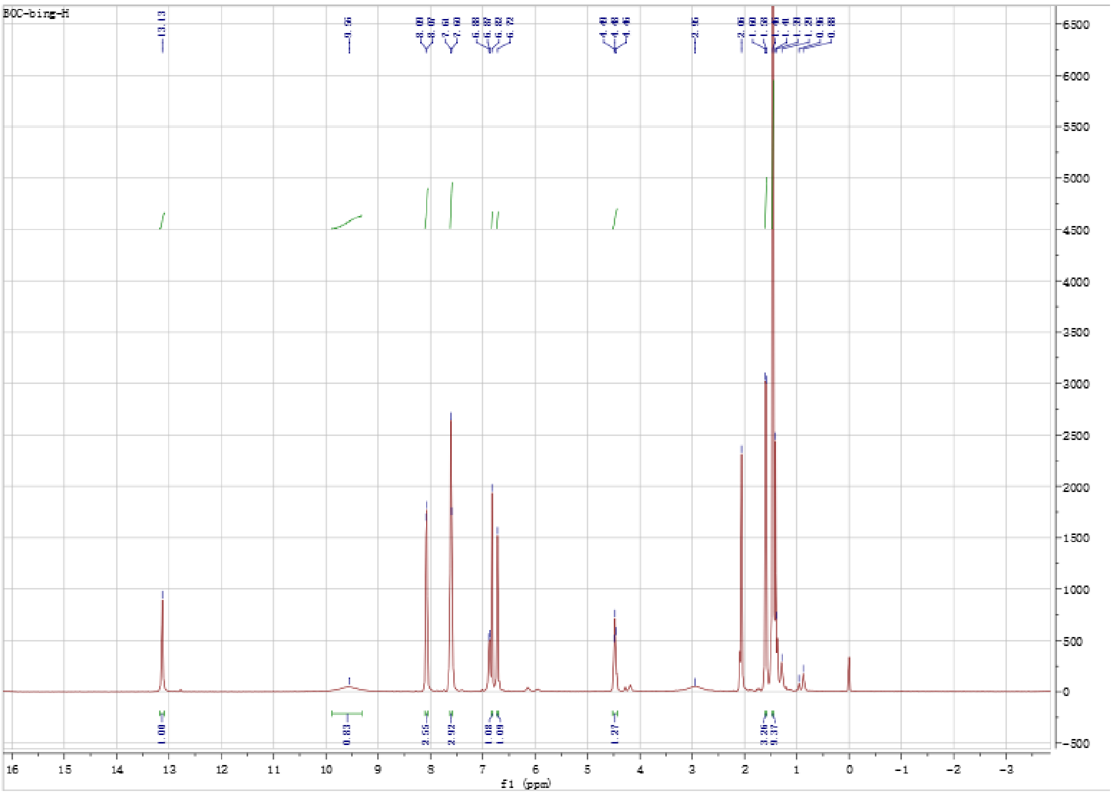

^{13}C NMR spectra of compound **14**

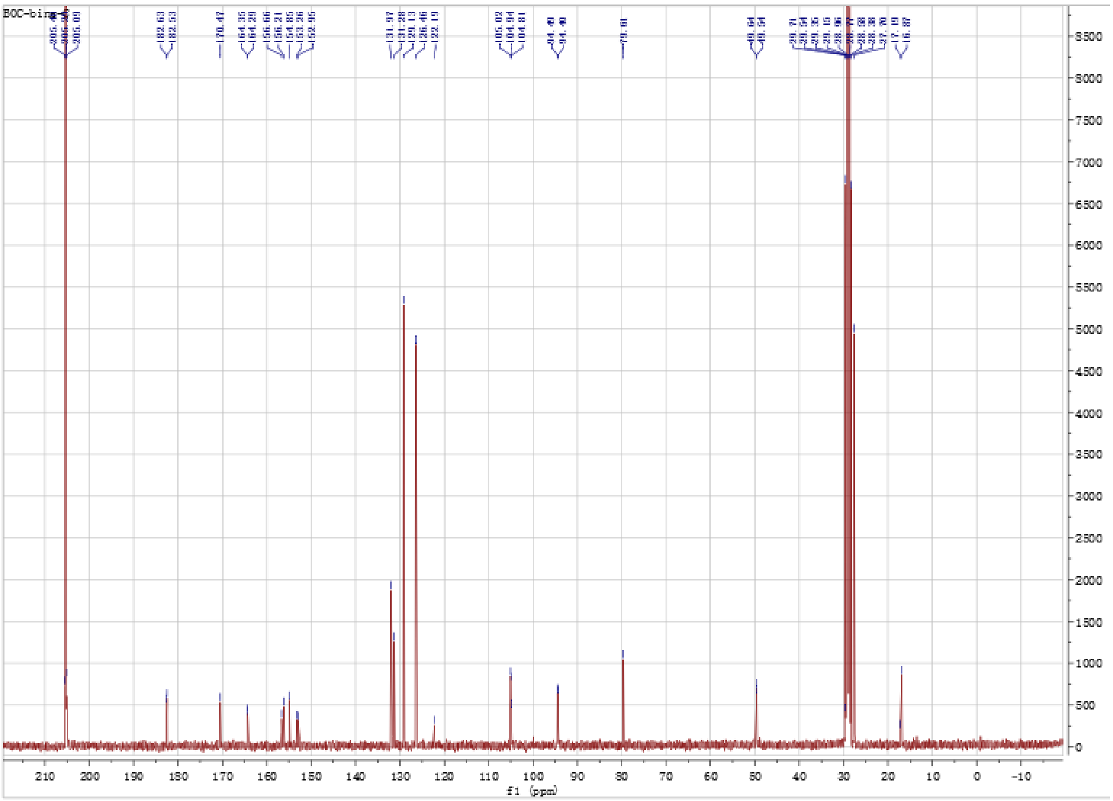

HR-MS of Compound **14**

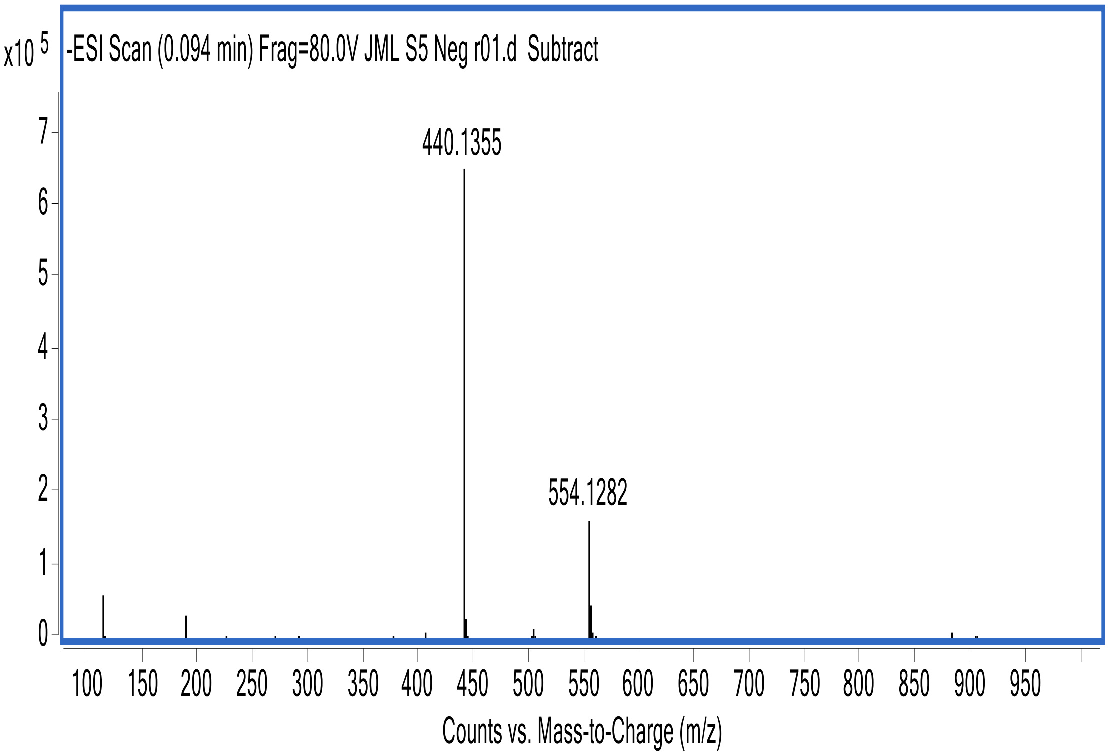

Compound **15**

^{1}H NMR spectra of compound **15**

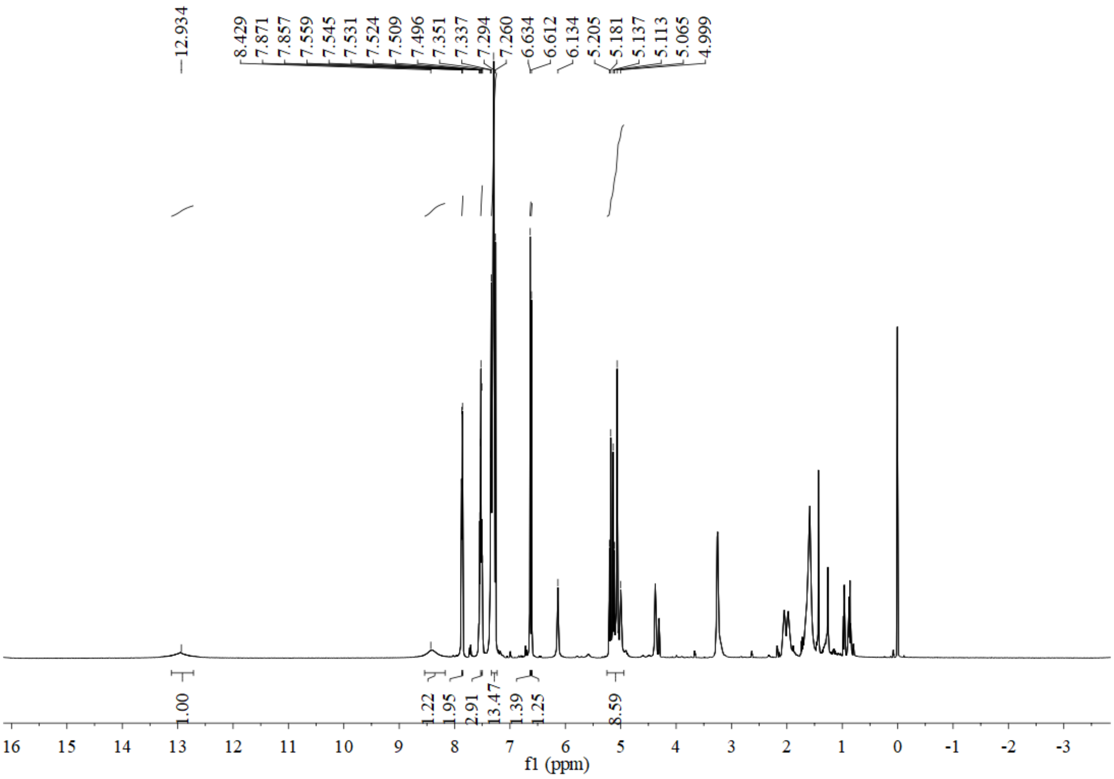

^{13}C NMR spectra of compound **15**

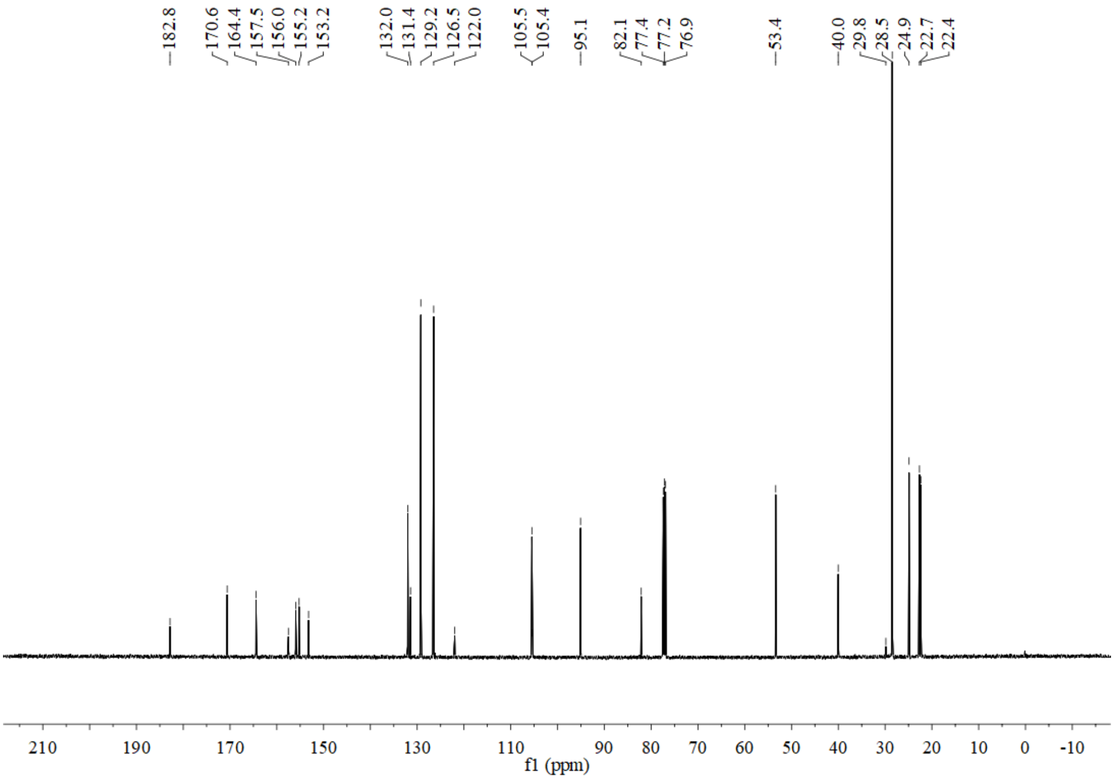

HR-MS of Compound **15**

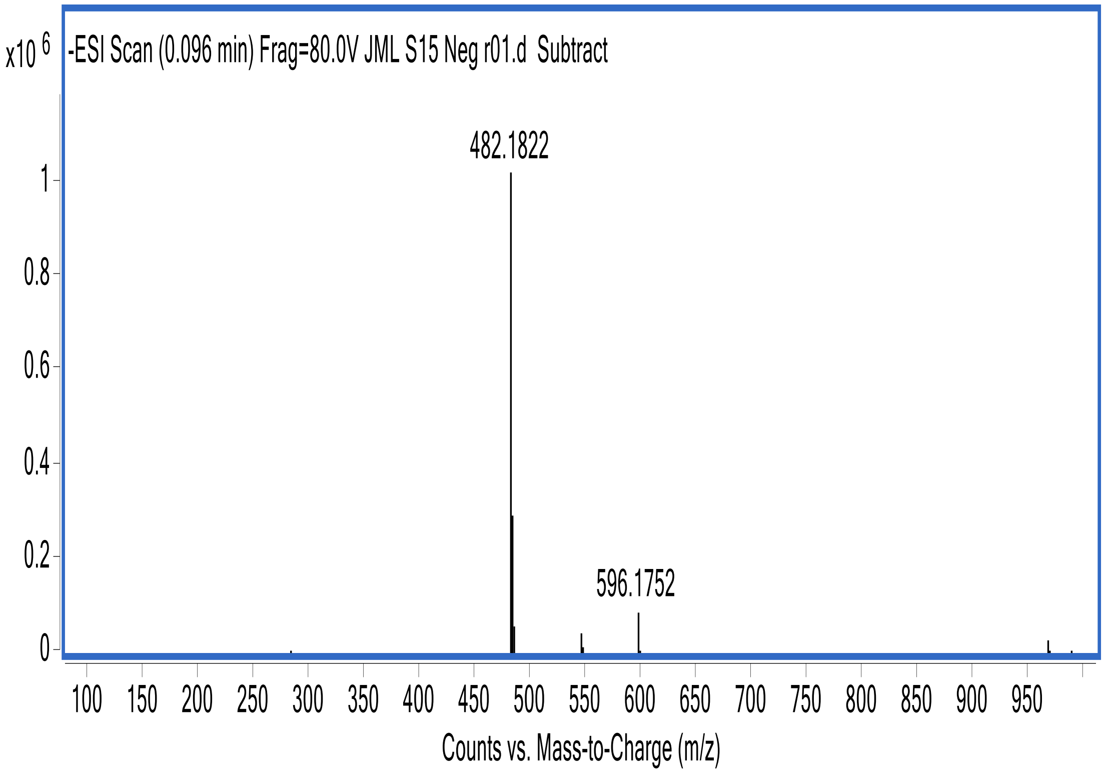

Supplement: Supplementary file 1 [file molecules-24-03647-s001.pdf]
